# Diversity and levels of endemism of the Bromeliaceae of Costa Rica – an updated checklist

**DOI:** 10.3897/phytokeys.29.4937

**Published:** 2013-11-11

**Authors:** Daniel A. Cáceres González, Katharina Schulte, Marco Schmidt, Georg Zizka

**Affiliations:** 1 Abteilung Botanik und molekulare Evolutionsforschung, Senckenberg Forschungsinstitut Frankfurt/Main, Germany; 2 Institut Ökologie, Evolution & Diversität, Goethe-Universität Frankfurt/Main, Germany; 3 Biodiversität und Klima Forschungszentrum (BiK-F), Frankfurt/Main, Germany; 4 Australian Tropical Herbarium & Center for Tropical Biodiversity and Climate Change, James Cook University, Cairns, Australia

**Keywords:** Central America, epiphytism, life-form, systematic diversity

## Abstract

An updated inventory of the Bromeliaceae for Costa Rica is presented including citations of representative specimens for each species. The family comprises 18 genera and 198 species in Costa Rica, 32 species being endemic to the country. Additional 36 species are endemic to Costa Rica and Panama. Only 4 of the 8 bromeliad subfamilies occur in Costa Rica, with a strong predominance of Tillandsioideae (7 genera/150 spp.; 75.7% of all bromeliad species in Costa Rica). 124 species (62.6%) grow exclusively epiphytic, additional 59 spp. (29.8%) are facultative epiphytes. The most diverse genus is *Werauhia*, with 59 species (29.8% of the Costa Rican bromeliad flora), followed by *Tillandsia* with 40 species (20.2%) and *Guzmania* with 28 spp. (8.6%).

## Abstract (es)

Es presentada una lista de chequeo actualizada de 18 géneros y 198 especies de Bromeliaceae conocidas de Costa Rica. Por consiguiente, las bromelias endémicas de Costa Rica, ahora comprenden 32 especies. Adicionalmente, 36 especies tienen un endemismo compartido con Panamá. Sólo 4 de las 8 subfamilias actualmente reconocidas ocurren en Costa Rica con fuerte predominio de Tillandsioideae (7 géneros/150 spp.; 75.7% del total de especies de bromelias de Costa Rica). 124 especies (62.6%) crecen como epífita, 59 especies adicionales (29.8%) son epifitas facultativas. El género mejor representado es *Werauhia*, con 59 especies (29.8% de la flora de bromelias de Costa Rica), seguido por *Tillandsia* con 40 spp. (20.2%) y *Guzmania* con 28 especies (8.6%).

## Introduction

Costa Rica is part of the hotspots of plant diversity in the Neotropics (Myers et al. 2000) with an estimated 8,249 vascular plant species (Hammel et al. 2004). Over the last century the number of scientifically documented plants for Costa Rica has increased considerably rendering the country one of the best studied in Mesoamerica. In Costa Rica, the Bromeliaceae belong to the families with highest species diversity in epiphytic habitats and thus contribute considerably to overall forest diversity.

Bromeliaceae comprise 3,172 species and 58 genera (Luther 2008), which are grouped in the 8 subfamilies Brocchinioideae, Lindmanioideae, Tillandsioideae, Hechtioideae, Navioideae, Pitcarnioideae, Puyoideae and Bromelioideae (Givnish et al. 2011). All bromeliads are restricted to the Neotropics, with the one exception of *Pitcairnia feliciana* from West Africa.

Based on the extensive revision of herbarium collections from Costa Rica, we present an updated checklist of Bromeliaceae for the country, and provide a brief analysis of systematic diversity, levels of endemism, distribution and life-form spectrum (epiphytic/terrestrial/saxicolous). Recent floristic work, in particular the revision of the diversity of Bromeliaceae of Panama (Cáceres González et al. 2011b) required the reassessment of endemicity of Costa Rican bromeliads. This led to considerable changes in the number of endemic bromeliad species recognized for Costa Rica.

## Materials and methods

Herbarium collections of Bromeliaceae from 23 herbaria (B, C, CR, DUKE, F, FR, GH, INB, K, LI, MICH, MO, NY, PMA, RM, SCZ, SEL, TEX, US, USJ, UMO, WS and WU; abbreviations after Thiers 2008) were studied. Altogether 4,523 herbarium specimens of Bromeliaceae from Costa Rica were revised.

For identification and extraction of biological information, the following references were used: Smith and Downs (1974, 1977, 1979), Utley (1994), Méndez-Estrada (1995), Grant (1995a, 1995b), Luther and Kress (1996), Grant and Morales (1996), Rossi et al. (1997), Morales (1999, 2000, 2003a, 2003b, 2003c, 2005, 2009), Morales and Alfaro (2003), Luther (2003), Cascante Marín et al. (2008), and Cáceres González et al. (2011a, 2011b). Further relevant references are given under the relevant species.

All species are listed below with information on their distribution and life form. Species endemic to Costa Rica are marked with an asterisk *. The endemic species reported by Morales (2003c) are marked with a “1”. Species endemic to Costa Rica and Panama are marked with a “2”.

Type specimens are annotated (holo = holotype, iso = isotype, lecto = lectotype, para = paratype). Additionally, a maximum of five revised herbarium collections are listed for each species, except in cases where the number of available collections was less than five.

The presented checklist includes indigenous taxa and only one cultivated and naturalized taxon (*Ananas comosus* (L.) Merr.). Subspecies, varieties, and forms are not included in the list. Species erroneously reported for Costa Rica are listed separately. Synonyms (syn.) are only included if found in the recent literature.

Nomenclature, generic delimitation and total species numbers for genera follow Luther (2008) and the International Plant Names Index (IPNI 2011).

## Results and discussion

### Bromeliaceae diversity in Costa Rica

According to our studies, Costa Rica harbours 18 genera and 198 species of Bromeliaceae, which represents 2.4% of the total angiosperm flora of the country (see species list [Appendix] and and Table 1). Thus, species diversity of the Costa Rican Bromeliaceae is one of the richest among the Central American countries. Of the 3,172 species and 58 genera recognized for the whole family (Luther 2008), 6.2% of the species and 31% of the generic diversity are represented in Costa Rica.

**Table 1.** Bromeliaceae of Costa Rica: species richness and endemism.

Subfamily & genus	Number of species	% of bromeliad flora	Number of endemic species	% of endemic species
**Tillandsioideae**				
*Catopsis*	11	5.6	1	3.1
*Guzmania*	28	14.1	4	12.5
*Mezobromelia*	1	0.5	0	0.0
*Racinaea*	5	2.5	0	0.0
*Tillandsia*	40	20.2	2	6.3
*Vriesea*	6	3.0	2	6.3
*Werauhia*	59	29.8	18	56.3
**Subtotal=7**	**150**	**75.7**	**27**	**84.5**
**Bromelioideae**				
*Aechmea*	17	8.6	0	0
*Ananas*	2	1.0	0	0
*Androlepis*	1	0.5	0	0
*Araeococcus*	1	0.5	0	0
*Billbergia*	1	0.5	0	0
*Bromelia*	3	1.5	0	0
*Greigia*	2	1.0	0	0
*Ronnbergia*	1	0.5	0	0
**Subtotal=8**	**28**	**14.1**	**0**	**0.0**
**Pitcairnioideae**				
*Pepinia*	1	0.5	1	3.1
*Pitcairnia*	17	8.6	3	9.3
**Subtotal=2**	**18**	**9.1**	**4**	**12.4**
**Puyoideae**				
*Puya*	2	1.0	1	3.1
**Subtotal=1**	**2**	**1.0**	**1**	**3.1**
**TOTAL=18**	**198**	**100.0**	**32**	**100.0**

The increase in species reported for Costa Rica in the recent past (e.g. Utley (1994): 169 species; Luther (1995): 192 spp.; and Morales (2003c): 195 spp) can mostly be attributed to the discovery and description of new species (Luther and Kress 1996: *Guzmania herrerae* H. Luther & W. J. Kress, *Guzmania scandens* H. Luther & W. J. Kress; Grant and Morales 1996: *Pitcairnia calcicola* J. R. Grant & J. F. Morales; Morales 1999: *Vriesea barii* J.F. Morales, *Vriesea simulans* J.F. Morales, *Vriesea haberii* J.F. Morales, *Vriesea osaensis* J.F. Morales, *Vriesea tiquirensis* J.F. Morales, *Vriesea vulcanicola* J.F. Morales). Nevertheless, there were several discrepancies between previous studies of the bromeliad flora for Costa Rica (e.g. Luther 1995; Morales and Alfaro 2003; Morales 2003c), which we were able to resolve. For example, Luther (1995) added *Tillandsia streptophylla* Scheidw. ex C. Morren as new for Costa Rica. Our attempt to trace the specimens on which such report was based was not successful, and perhaps for this reason, Morales (2003c) refrained from including *Tillandsia streptophylla* in his list of bromeliads of Costa Rica. Nevertheless, we were able to verify the presence of the species based on recently collected specimens revised by us, thus *Tillandsia streptophylla* is again included in our checklist (see Appendix).

According to Morales (2009), *Aechmea penduliflora* André, formerly reported for Costa Rica, does not exist in the country. Morales (2009) refers to misidentified collections (*Espinoza 94*, INB, MO; *McPherson 8533*, MO; *Rueda & Mendoza 17152*, MO), which represent *Aechmea angustifolia* Poepp. & Endl. However, other specimens from Costa Rica revised by us represent *Aechmea penduliflora* (see Appendix), thus we included the species in our list.

Morales (2003c) included *Catopsis werckleana* Mez in the synonymy of *Catopsis nutans* (Sw.) Griseb. However, we accept *Catopsis werckleana* as a separate species which is documented from Costa Rica and thus included in our list.

### Species erroneously reported or with name unresolved for Costa Rica

The records of *Catopsis wawranea*, cited by Smith and Downs (1977), Utley (1994) and Luther (1995) are based on the collection *Werckle s.n.* (B). We determined this collection to be *Catopsis wangerinii* Mez & Wercklé, therefore *Catopsis wawranea* had to be excluded from the list.

*Werauhia cooperiana* J. F. Morales, listed and described by Morales (2003c), is based on the specimen *Morales & Soto 7700* (INB, n.v.). In this publication this species is specified as “in press” (Morales 2003c: 360) and according to The Plant List (2010) “this name is unresolved” and not listed by Luther (2008). Since we did not study the relevant specimen, *Werauhia cooperiana* is excluded from this checklist.

Altogether, 195 species of our list (198 spp.) are also documented in Morales (2003c), Morales (2005, 2009) and Morales and Alfaro (2003). In total, five species (*Catopsis werckleana*, *Tillandsia streptophylla*, *Tillandsia rhomboidea*, *Tillandsia guatemalensis*, *Werauhia anitana*) had to be added and two (*Tillandsia acostae*, *Guzmania mitis*) were excluded based on our revision of the herbarium material.

## Taxonomic diversity

Among the four subfamilies of Bromeliaceae represented in Costa Rica, Tillandsioideae are the most diverse (7 genera/150 spp.; 75.7% of all Costa Rican bromeliad species), followed by Bromelioideae (8/28; 14.1%), Pitcairnioideae s.str. (2/18; 9.1%) and Puyoideae (1/2; 1%) (Table 1). A similar dominance of Tillandsioideae is also found in Mexico (Espejo-Serna et al. 2004), Panama (Cáceres González et al. 2011b), Colombia (Holst 1994), Ecuador (Holst 1994), Peru (Holst 1994), and Bolivia (Krömer et al. 1999).

At the generic level, *Werauhia* is the most diverse group in Costa Rica with 59 species (29.8% of the bromeliad flora). The genus has its centre of diversity in Costa Rica and Panama. Second in species richness is *Tillandsia* (40 spp., 20.2%), followed by *Guzmania* (28 spp., 14.1%), *Pitcairnia* (17 spp., 8.6%), and *Aechmea* (17 spp., 8.6%) (Table 1).

### Life-form

**Figure 1.**
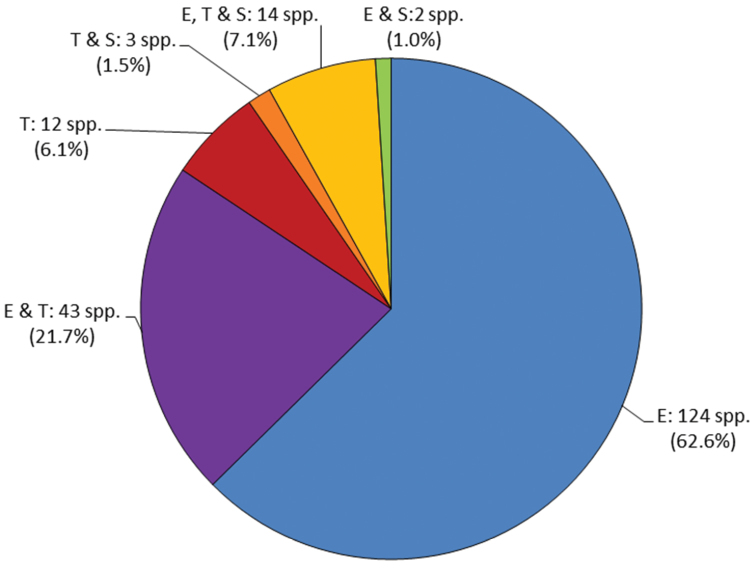
Bromeliaceae of Costa Rica: life form spectrum. **E**: epiphytic, **T**: terrestrial, **S**: saxicolous.

Of the 198 bromeliad species reported for Costa Rica, 124 (62.6%) grow epiphytically (e.g. *Catopsis nutans*, *Tillandsia caput-medusae*, *Werauhia osaensis*) and 12 (6.1%) as terrestrials (e.g. *Aechmea magdalenae*, *Greigia columbiana*). Alltogether 43 spp. (21.7%) can be found growing epiphytic or terrestrial (e.g. *Guzmania plicatifolia*, *Werauhia kupperiana*), 2 species (1.0%) were found growing both, epiphytic and saxicolous (*Pitcairnia saxicola*, and *Tillandsia brachycaulos*). Only 3 species (1.5%) were found exclusively growing in soil or on rocks (*Pitcairnia calcicola*, *Pitcairnia halophila*, and *Puya floccosa*). The number of species that can be found as epiphytes, terrestrials and/or saxicoles adds up to 14 (7.1%) (Fig. 1). The high number of epiphytes in the bromeliad flora can be explained by the dominance of different types of tropical forests in the natural vegetation of the country (Cáceres González et al. 2011b).

### Endemism

Previously, 44 species were regarded to be endemic to Costa Rica (Morales 2003c), equalling 22.6% of the total bromeliad flora known at that time (195 spp.). With the new records of bromeliad species recently reported for Panama (Cáceres González et al. 2011b) and the results presented here, the number of bromeliads endemic to Costa Rica is reduced to 32 species (16.2%). A considerable number of species previously regarded as endemic to Costa Rica is now known to occur also in Panama. In total, 36 species (18.2%) have a shared endemism with Panama (see Appendix and Table 1).

In Costa Rica, the level of endemism in the family Bromeliaceae is distributed among the subfamilies as follows: the Tillandsioideae comprise the majority of endemic species (27 species (84.5%)), followed by Pitcairnioideae with 4 species (12.4%) and Puyoideae with 1 species (3.1%) (Table 1).

Many bromeliad species with endemism shared between Panama and Costa Rica have been collected principally in the Cordillera de Talamanca of both countries.

The transborder Parque Internacional La Amistad (PILA), including nearly 1,940 km^2^ of Costa Rica and 2,070 km^2^ of Panama was founded in 1988 and declared a World Heritage Site in 1990. It is an important contribution to the conservation of biodiversity in this hotspot. Considering that the diversity of epiphytic bromeliads of Costa Rica and Panama is a good indicator for overall diversity in forests, additional protected areas in this mountainous region with exceptionally high geo- and biodiversity are highly desirable.

## Appendix

### AECHMEA

***Aechmea angustifolia*** Poepp. & Endl., Nov. Gen. Sp. Pl. 2: 43 (1838).

**Distribution.** Nicaragua, Costa Rica, Panama, Colombia, Ecuador, Peru, Bolivia, Brazil, and Venezuela.

**Representative Collections—COSTA RICA.** *Pittier 9426* (B); 9 Apr 1983, *Liesner 14163* (INB, SEL); 20 Feb 1991, *Till 7053* (CR); 1 Feb 1996, *Hammel 20142* (INB); 6 May 1998, *Morales 6422* (INB).

**Life form.** Epiphyte.

***Aechmea aquilega*** (Salisb.) Griseb., Fl. Brit. W. I.: 592 (1864).

**Distribution.** Costa Rica, Brazil, Suriname, Guianas, Venezuela, and Trinidad and Tobago.

**Representative Collections—COSTA RICA.** 19 Jun 1874, *Kuntze 2113* (CR, n.v.; K, n.v.).

**Life form.** Epiphyte.

***Aechmea bracteata*** (Sw.) Griseb., Fl. Brit. W. I.: 592 (1864).

**Distribution.** Mexico, Guatemala, Belize, Honduras, Nicaragua, Costa Rica, and Panama.

**Representative Collections—COSTA RICA.** 25 Feb 1933, *Valerio 375* (CR); 10 Mar 1994, *Huber 355* (CR); 28 May 1995, *Grant 2348* (INB); 10 Feb 1997, *Moraga 863* (CR, INB); 11 Apr 2005, *Morales 12758* (INB).

**Life form.** Epiphyte.

***Aechmea castelnavii*** Baker, Handb. Bromel.: 39 (1889).

**Distribution.** Costa Rica, Colombia, Bolivia, Brazil, and Venezuela.

**Representative Collections—COSTA RICA.** 17 Jan 1991, *Grant 91-01478* (INB); 13 May 1995, *Jiménez 1827* (INB); 5 Apr 2000, *Acosta 807* (INB); 3 Apr 1997, *Morales 6140* (INB); 4 Mar 2001, *Morales 7676* (INB).

**Life form.** Epiphyte.

***Aechmea dactylina*** Baker, J. Bot. 17: 161 (1879).

**Distribution.** Nicaragua, Costa Rica, Panama, Colombia, and Ecuador.

**Representative Collections—COSTA RICA.** 4 Jan 1896, *Tonduz 9985* (CR); 1 Aug 1949, *Holm 721* (CR); 13 May 1989, *Kress 2720* (INB, SEL); 27 Jul 1995, *Lépiz 613* (INB); 21 Aug 2003, *Morales 9457* (INB).

**Life form.** Epiphyte.

***Aechmea lingulata*** (L.) Baker, J. Bot. 17: 164 (1879).

**Distribution.** Costa Rica, Panama, Brazil, Guianas, Venezuela, Trinidad, Tobago, and Jamaica.

**Representative Collections—COSTA RICA.** *Oersted 24* (C, n.v.), based on Smith & Downs (1979).

**Life form.** Epiphyte.

***Aechmea lueddemanniana*** (K. Koch) Brongn. ex Mez, Pflanzenr., IV, 32: 120 (1934).

**Distribution.** Mexico, Belize, Honduras, Nicaragua, and Costa Rica.

**Representative Collections—COSTA RICA.** 27 Mar 1989, *Haber 9169* (INB); 2 Jun 1990, *Varela 11* (INB); 23 Mar 1994, *Morales 2555* (INB); 29 Jan 1995, *Rivera 1005* (INB); 12 Feb 1999, *Alvarado 71* (INB).

**Life form.** Epiphyte.

***Aechmea magdalenae*** (André) André ex Baker, Handb. Bromel.: 65 (1889).

**Distribution.** Mexico, Guatemala, Belize, El Salvador, Honduras, Nicaragua, Costa Rica, Panama, Colombia, Ecuador, and Venezuela.

**Representative Collections—COSTA RICA.** 3 Jan 1896, *Tonduz 9897* (CR); 21 Jul 1949, *Holm & Iltis 439* (NY); 24 Mar 1973, *Burger 8869* (CR); 2 Jul 1994, *Lépiz 452* (CR); 25 Jan 2000, *Acosta 280* (CR, INB).

**Life form.** Terrestrial.

***Aechmea mariae-reginae*** H. Wend., Hamb. Gartenz. 19: 32 (1863).

**Distribution.** Nicaragua, Costa Rica, and Panama.

**Representative Collections—COSTA RICA.** *Wercklé 16202* (B); 8 Jun 1976, *Utley 5114* (CR); 14 Feb 1982, *Burger & Gomez 11818* (PMA); 2 Sep 1993, *Morales 1640* (INB, NY); 3 Aug 2006, *Cascante 1598* (CR).

**Life form.** Epiphyte (rarely terrestrial).

***Aechmea mexicana*** Baker, J. Bot. 17: 165 (1879).

**Distribution.** Mexico, Guatemala, Nicaragua, Costa Rica, Panama, Colombia, and Ecuador.

**Representative Collections—COSTA RICA.** 3 Jan 1896, *Tonduz 10074* (CR); Mar 1947, *Buchanan 546* (NY); 19 Dec 1988, *Bello 624* (CR, INB); 21 Feb 1995, *Morales 3508* (CR, INB); 12 May 1999, *Estrada 2214* (CR).

**Life form.** Epiphyte.

***Aechmea nudicaulis*** (L.) Griseb., Fl. Brit. W. I.: 593 (1864).

**Distribution.** Mexico, Guatemala, Honduras, Nicaragua, Costa Rica, Panama, Colombia, Ecuador, Brazil, and Dominican Republic.

**Representative Collections—COSTA RICA.** 8 Dec 1973, *Utley 510* (CR); 25 Nov 1982, *McDowell 941* (SCZ); 3 Dec 1993, *Morales 2135* (CR, INB); 6 Mar 1998, *Vargas 37* (CR, INB); 27 Sep 2000, *Acosta 2757* (INB).

**Life form.** Epiphyte.

***Aechmea penduliflora*** André, Rev. Hort. 60: 563 (1888).

**Distribution.** Nicaragua, Costa Rica, Panama, Colombia, Ecuador, Peru, Bolivia, and Venezuela.

**Representative Collections—COSTA RICA.** 1894, *Friedrichsthal 4962* (B); Apr 1894, *J. D. Smith 4962* (US, n.v.); Mar 1924, *Standley 37556* (US, n.v.); 29 Feb 1956, *Schubert 1126* (US, n.v.); 3 Dec 1980, *Waggoner s.n.* (SEL).

**Life form.** Epiphyte.

**^1,2^*Aechmea pittieri*** Mez, Monogr. Phan. 9: 231 (1896).

**Distribution.** Costa Rica and Panama.

**Representative Collections—COSTA RICA.** Jan 1892, *Pittier 6609* (B); Jan 1908, *Wercklé 17422* (B); 26 Jan 1967, *Burger & Matta 4673* (NY); 9 Dec 2004, *Soto 454* (INB); 27 Jan 2007, *Morales 15388* (INB).

**Life form.** Epiphyte (rarely terrestrial).

***Aechmea pubescens*** Baker, J. Bot. 17: 135 (1879).

**Distribution.** Honduras, Nicaragua, Costa Rica, Panama, Colombia, and Venezuela.

**Representative Collections—COSTA RICA.** 4 Apr 1930, *Brenes 1293* (CR); 26 May 1973, *Ocampo 403* (CR); 28 May 1988, *Hammel 16870* (INB); 27 May 2003, *Holst 8654* (SEL); 14 Jan 2005, *Solano 1707* (INB).

**Life form.** Epiphyte (occasionally terrestrial).

***Aechmea tillandsioides*** (Mart. ex Schult. f.) Baker, J. Bot. 17: 134 (1879).

**Distribution.** Mexico, Guatemala, Belize, El Salvador, Honduras, Nicaragua, Costa Rica, Panama, Colombia, Ecuador, Peru, Bolivia, Brazil, Guianas, and Venezuela.

**Representative Collections—COSTA RICA.** 2 May 1951, *León 3409* (CR); 26 Apr 1973, *Burger & Gentry 9287* (NY); 10 Jul 1991, *Delprete 5199* (CR, INB, MO, NY; TEX, n.v.); 21 Mar 1997, *Morales 6126* (CR, INB); 29 Oct 2000, *Gómez-Laurito 13418* (CR).

**Life form.** Epiphyte.

***Aechmea tonduzii*** Mez & Pittier ex Mez, Bull. Herb. Boissier, II, 3: 132 (1903).

**Distribution.** Costa Rica, Panama, Colombia, and Ecuador.

**Representative Collections—COSTA RICA.** Mar 1893, *Tonduz 7684* (B, holo); 24 Jan 1989, *Grayum 9292* (INB); 19 Jun 1995, *Chacón 263* (CR, USJ); 26 May 2003, *Clark 153* (SEL); 2 Mar 2005, *Morales 12277* (INB).

**Life form.** Epiphyte and terrestrial.

***Aechmea veitchii*** Baker, Bot. Mag. 103: t. 6329 (1877).

**Distribution.** Costa Rica, Panama, Colombia, Ecuador, and Peru.

**Representative Collections—COSTA RICA.** 12-17 Dec 1969, *Burger & Liesner 6775* (PMA); 2 Oct 1983, *Chacón 1442* (CR); 20 Jul 1994, *Lépiz 498* (CR, INB); 23 Mar 2000, *Acosta 699* (CR, INB); 25 Oct 2007, *Santamaría 6642* (INB).

**Life form.** Epiphyte and terrestrial.

***ANANAS***

***Ananas ananassoides*** (Baker) L. B. Sm., Bot. Mus. Leafl. 7: 79 (1939).

**Distribution.** Costa Rica, Panama, Colombia, Ecuador, Peru, Bolivia, Paraguay, Brazil, and Venezuela.

**Representative Collections—COSTA RICA.** 15 Nov 1990, *Skotak 1* (INB); 1 Feb 1991, *Skotak s.n.* (CR); 6 Feb 2000, *Aguilar 5807* (INB); 25 Aug 2001, *Hammel 22407* (INB); 25 Apr 2004, *Morales 10477* (INB).

**Life form.** Terrestrial.

***Ananas comosus*** (L.) Merr., Interpr. Herb. Amboin.: 133 (1917).

**Distribution.** Mexico, Guatemala, Belize, Honduras, Nicaragua, Costa Rica, Panama, Colombia, Ecuador, Peru, Bolivia, Brazil, Venezuela, Dominican Republic, and United States of America.

**Representative Collections—COSTA RICA.** 7 Jan 1992, *Grant 92-1773* (CR, INB); 25 Mar 1998, *Lépiz 255* (INB); 19 Sep 2002, *Frances 19* (CR); 25 Mar 2005, *Morales 12423* (INB); 8 Apr 2006, *Morales 13793* (INB).

**Life form.** Terrestrial. Cultivated and naturalized.

***ANDROLEPIS***

***Androlepis skinneri*** Brongn. ex Houllet, Rev. Hort. 42: 12 (1870).

**Distribution.** Mexico, Guatemala, Belize, Nicaragua, and Costa Rica.

**Representative Collections—COSTA RICA.** 16 Oct 1933, *Dodge 4864* (INB); 7 Aug 1949, *Holm 935* (CR); 5 y 6 Jun 1967, *Burger & Matta 4214* (NY); 20 Dec 1969, *Burger 6987* (CR); 1 Dec 1988, *Robles 2194* (INB).

**Life form.** Epiphyte.

***ARAEOCOCCUS***

***Araeococcus pectinatus*** L. B. Sm., Contr. Gray Herb. 95: 41 (1931).

**Distribution.** Costa Rica, Panama, and Colombia.

**Representative Collections—COSTA RICA.** 30 May 1950, *Allen 5558* (NY); 11 Jul 1987, *Gómez-Laurito 11577* (CR, INB); 28 May 1988, *Hammel 16873* (CR); 10 Sep 1996, *Croat 79160* (SEL); 2 Mar 2005, *Morales 12190* (INB).

**Life form.** Epiphyte.

***BILLBERGIA***

***Billbergia macrolepis*** L. B. Sm., Contr. Gray Herb. 114: 3 (1936).

**Distribution.** Costa Rica, Panama, Ecuador, and Venezuela.

**Representative Collections—COSTA RICA.** 1 Jan 1892, *Pittier 6608* (INB); 3 Mar 2000, *Hammel 22122* (INB); 25 May 2003, *Hammel 22777* (INB); 13 Jan 2010, *Hammel 25511* (INB).

**Life form.** Epiphyte.

***BROMELIA***

***Bromelia hemisphaerica*** Lam., Encycl. 1: 145 (1783).

**Distribution.** Mexico, Nicaragua, Costa Rica, and Panama.

**Representative Collections—COSTA RICA.** *Wercklé s.n.* (B, lecto); 9 Jun 1992, *Grant 1912* (INB); 28 May 1994, *Morales 2808* (INB, SEL); 22 Jan 1999, *Hurtado 47* (INB); 14 Sep 2003, *Morales 9876* (INB).

**Life form.** Terrestrial.

***Bromelia karatas*** L., Sp. PI. 1: 285 (1753).

**Syn.:** *Bromelia plumieri* (E. Morren) L.B. Sm., Phytologia 15: 173 (1967).

**Distribution.** Mexico, Guatemala, Belize, Honduras, Nicaragua, Costa Rica, Panama, Colombia, Ecuador, Brazil, Suriname, Venezuela, and Dominican Republic.

**Representative Collections—COSTA RICA.** 22 Jun 1977, *Liesner 2347* (CR); 28 Jun 1991, *Hammel 18231* (CR, INB, SEL); 23 Nov 1993, *Morales 2077* (INB); 10 Mar 2000, *Hammel 22195* (INB); 24 Sep 2004, *Acosta 3470* (INB).

**Life form.** Terrestrial.

***Bromelia pinguin*** L., Sp. Pl.: 285 (1753).

**Distribution.** Mexico, Guatemala, Belize, El Salvador, Honduras, Nicaragua, Costa Rica, Panama, Colombia, Ecuador, Venezuela, Puerto Rico, Dominican Republic, Haiti, and Jamaica.

**Representative Collections—COSTA RICA.** 2 Jun 1932, *Brenes 15916* (CR); 2 Aug 1971, *Burger 7861* (CR); 13 Jul 1985, *Worthington 13541* (NY); 4 Apr 2001, *Morales 7941* (CR, INB); 18 Mar 2005, *Santamaría 1063* (INB).

**Life form.** Terrestrial.

***CATOPSIS***

***Catopsis berteroniana*** (Schult. & Schult. f.) Mez, Monogr. Phan. 9: 621 (1896).

**Distribution.** Mexico, Guatemala, Belize, Honduras, Nicaragua, Costa Rica, Panama, Colombia, Ecuador, Brazil, Guianas, Venezuela, Dominican Republic, Jamaica, and United States of America.

**Representative Collections—COSTA RICA.** 19 Jan 1994, *Morales 3341* (INB); 10 May 1995, *Morales 4095* (INB).

**Life form.** Epiphyte.

***Catopsis hahnii*** Baker, J. Bot. 25: 175 (1887)

**Distribution.** Mexico, Guatemala, Belize, El Salvador, Honduras, Nicaragua, and Costa Rica.

**Representative Collections—COSTA RICA.** 1847?, *Oersted 18* (B); 11 Oct 1999, *Morales 8455* (INB); 11 feb 2005, *Morales 11984* (INB).

**Life form.** Epiphyte.

***Catopsis juncifolia*** Mez & Wercklé, Bull. Herb. Boissier, II, 4: 1124 (1904).

**Distribution.** Nicaragua, Costa Rica, and Panama.

**Representative Collections—COSTA RICA.** *Wercklé 133* (B, holo); 15 Oct 1988, *Herrera 2190* (CR, INB); 17 Apr 1994, *Lépiz 289* (INB); 12 May 2005, *Morales 12961* (INB); 23 May 2007, *Zamora 3986* (INB).

**Life form.** Epiphyte.

***Catopsis micrantha*** L. B. Sm., Ann. Missouri Bot. Gard. 30: 83 (1943).

**Syn.:** *Catopsis pedicellata* L. B. Sm., Contr. Gray Herb. 154: 34 (1945).

**Distribution.** Guatemala, Costa Rica, and Panama.

**Representative Collections—COSTA RICA.** 19 Jan 1994, *Morales 3342* (INB); 18 Nov 2004, *Morales 11616* (INB).

**Life form.** Epiphyte.

***Catopsis morreniana*** Mez, Mongr. Phan. 9: 628 (1896).

**Distribution.** Mexico, Guatemala, Belize, Honduras, Nicaragua, and Costa Rica.

**Representative Collections—COSTA RICA.** *Wercklé 135* (B); 15 Oct 1987, *Herrera 878* (CR, INB); 23 Jul 1990, *Bello 2332* (CR, INB); 30 Jun 1994, *Kress 94-4059* (CR); 23 Abr 2006, *Morales 13862* (INB).

**Life form.** Epiphyte.

***Catopsis nitida*** (Hook.) Griseb., Fl. Brit. W. I.: 599 (1864).

**Distribution.** Guatemala, Belize, Honduras, Nicaragua, Costa Rica, Panama, and Dominican Republic.

**Representative Collections—COSTA RICA.** 1 Jan 1901, *Wercklé 16191* (CR; US, n.v.); 23 May 1969, *Gómez 2215* (NY); 22 Jul 1990, *Luther 2807* (CR); 13 Aug 1993, *Palaci 1206* (CR, INB); 5 Mar 2008, *Cascante 1860* (CR).

**Life form.** Epiphyte.

***Catopsis nutans*** (Sw.) Griseb., Fl. Brit. W. I.: 599 (1864).

**Distribution.** Mexico, Guatemala, El Salvador, Honduras, Nicaragua, Costa Rica, Panama, Colombia, Ecuador, Venezuela, Dominican Republic, and United States of America.

**Representative Collections—COSTA RICA.** 24 Jun 1943, *Quirós 1141* (CR); 16 Dec 1973, *Utley 536* (CR); 22 Feb 1990, *Grant 90-833* (CR); 18 Jan 1992, *Ramírez 94-144* (USJ); 1 Mar 2007, *Monro 5729* (INB).

**Life form.** Epiphyte.

***Catopsis paniculata*** E. Morren, Phytologia 15: 179 (1967).

**Distribution.** Mexico, Guatemala, Honduras, Nicaragua, and Costa Rica.

**Representative Collections—COSTA RICA.** 19 Apr 1908, *Maxon 67* (NY); 30 Jul 1967, *Lent 1155* (CR); 22 Jun 1990, *Luther 2806* (CR, SEL); 30 Dec 1993, *Morales 2173* (INB, SEL); 22 Jul 2008, *Cascante 1983* (CR).

**Life form.** Epiphyte.

***Catopsis sessiliflora*** (Ruiz & Pav.) Mez, Monogr. Phan. 9: 625 (1896).

**Distribution.** Mexico, Guatemala, Belize, Honduras, Nicaragua, Costa Rica, Panama, Colombia, Ecuador, Peru, Bolivia, Brazil, Venezuela, and Puerto Rico.

**Representative Collections—COSTA RICA.** Jul 1936, *Skutch 2771* (NY); 6 Jul 1977, *Liesner 2969* (CR); 9 Sep 1996, *Hammel 20439* (CR, INB); 28 May 2005, *Santamaría 2165* (INB); 8 Mar 2008, *Morales 16251* (INB).

**Life form.** Epiphyte.

***Catopsis wangerinii*** Mez & Wercklé, Bull. Herb. Boissier, II, 4: 1126 (1904).

**Distribution.** Guatemala, El Salvador, Nicaragua, Costa Rica, and Panama.

**Representative Collections—COSTA RICA.** *Wercklé 105* (B, holo); 19 Apr 1908, *Maxon 65* (NY); 30 Sep 1994, *Fernández 1387* (CR, INB); 6 Feb 1998, *Till 15001* (SEL); 26 Abr 2008, *Hammel 24723* (INB).

**Life form.** Epiphyte.

****Catopsis werckleana*** Mez, Bull. Herb. Boissier, II, 4: 1125 (1904).

**Distribution.** Costa Rica.

**Representative Collections—COSTA RICA.** 1 Dec 1902, *Wercklé 65* (B, holo; MO, photo); Mar 1926, *Standley & Torres 51655* (US, n.v.); Apr 1983?, *Liesner & Judziewicz 14371* (MO, n.v.) (Utley 1994: 146).

**Life form.** Epiphyte.

***GREIGIA***

***Greigia columbiana*** L. B. Sm., Contr. Gray Herb. 98: 7 (1932).

**Distribution.** Costa Rica, Panama, and Colombia.

**Representative Collections—COSTA RICA.** 30 Jul 1962, *Lee 761* (CR); 24 Feb 1991, *Till 7105* (INB); 30 May 1995, *Grant 2335* (INB, SEL); 20 Jul 2000, *Alfaro 3235* (INB); 15 Jun 2005, *Morales 13138* (INB).

**Life form.** Terrestrial.

**^2^*Greigia sylvicola*** Standl., J. Wash. Acad. Sci. 17: 160 (1927).

**Distribution.** Costa Rica and Panama.

**Representative Collections—COSTA RICA.** 24 Feb 1977, *Dwyer 1218* (CR); 20 Nov 1988, *Kappelle 3676* (CR); 16 Apr 1991, *Haber 10691* (INB, SEL); 23 Mar 2000, *Acosta 690* (CR, INB, NY); 15 Jun 2005, *Morales 13127* (INB).

**Life form.** Terrestrial.

***GUZMANIA***

***Guzmania angustifolia*** (Baker) Wittm., Bot. Jahrb. Syst. 11: 62 (1889).

**Distribution.** Nicaragua, Costa Rica, Panama, Colombia, and Ecuador.

**Representative Collections—COSTA RICA.** 14 Jun 1938, *Smith 779* (NY); 6 Dec 1983, *Zamora 435* (CR); 19 Jun 1993, *Rivera 2240* (CR, SEL); 12 Feb 1994, *Herrera 6841* (USJ); 9 Mar 2008, *Morales 16323* (INB).

**Life form.** Epiphyte.

***^1^*Guzmania blassii*** Rauh, J. Bromeliad Soc. 33: 66 (1983).

**Distribution.** Costa Rica.

**Representative Collections—COSTA RICA.** 13 May 1989, *Kress 2722* (INB; SEL; US, n.v.); 11 Jul 1989, *Waggoner s.n.* (SEL); 3 Jun 1995, *Morales 4294* (INB); 26 May 2003, *Clark 144* (SEL); 25 Jul 2007, *Rodríguez 11309* (INB).

**Life form.** Epiphyte.

**^2^*Guzmania circinnata*** Rauh, Trop. Subtrop. Pflanzenwelt 60: 48 (1987).

**Distribution.** Costa Rica and Panama.

**Representative Collections—COSTA RICA.** 24 Sep 1991, *Skotak s.n.* (SEL); 24 Sep 1991, *Skotak 2* (INB).

**Life form.** Epiphyte.

***Guzmania compacta*** Mez, Monogr. Phan. 9: 947 (1896).

**Distribution.** Costa Rica and Ecuador.

**Representative Collections—COSTA RICA.** *Wercklé 17288* (B, lecto); 19 Jan 1991, *Rivera 998* (CR, INB); 8 Feb 1992, *Ingram 1300* (SEL); 29 Jan 1995, *Chavarría 1165* (INB); 18 Jan 2002, *Chaves 1402* (INB).

**Life form.** Epiphyte.

****Guzmania condensata*** Mez & Wercklé, Bull. Herb. Boissier, II, 3: 228 (1903).

**Distribution.** Costa Rica.

**Representative Collections—COSTA RICA.** 1 Jan 1901, *Wercklé 16198* (B, holo; CR, NY); 21 Apr 1983, *Liesner 14527* (CR, INB); 26 Jul 1990, *Luther s.n.* (SEL); 15 Mar 1994, *Morales 2490* (CR, INB); 23 Aug 2007, *Cascante 1806* (CR).

**Life form.** Epiphyte (rarely terrestrial).

***Guzmania coriostachya*** (Griseb.) Mez, Monogr. Phan. 9: 914 (1896).

**Distribution.** Costa Rica, Panama, Colombia, Ecuador, and Venezuela.

**Representative Collections—COSTA RICA.** 1904, *Wercklé 78* (B, lecto); 26 May 1973, *Ocampo 401* (CR); 10 Feb 1992, *Ingram 1309* (SEL); 8 Jun 2001, *Cascante 1509* (CR); 27 Jun 2004, *Morales 10845* (INB).

**Life form.** Epiphyte.

***Guzmania desautelsii*** Read & L. B. Sm., J. Brom. Soc. 33(1): 17 (1983).

**Distribution.** Nicaragua, Costa Rica, Panama, and Venezuela.

**Representative Collections—COSTA RICA.** 17 Sep 1978, *Burger 11130* (CR); 4 Mar 1991, *Rivera 1146* (CR, INB); 4 Apr 1995, *Moraga 75* (INB); 11 Feb 2000, *Mora 836* (INB); 9 Mar 2008, *Morales 16344* (INB).

**Life form.** Epiphyte (rarely terrestrial).

***Guzmania dissitiflora*** (André) L. B. Sm., Contr. Gray Herb. 104: 74 (1934).

**Distribution.** Costa Rica, Panama, Colombia, and Ecuador.

**Representative Collections—COSTA RICA.** *Brenes s.n.* (NY); 27 Aug 1967, *Lent 1216* (NY); 7 Dec 1982, *Gómez 19224* (NY); 16 Jan 1991, *Bittner 277* (INB); 31 Jan 1991, *Bittner 413* (INB).

**Life form.** Epiphyte.

***Guzmania donnellsmithii*** Mez ex Donn. Sm., Bot. Gaz. 35: 9 (1903).

**Distribution.** Nicaragua, Costa Rica, Panama, and Ecuador.

**Representative Collections—COSTA RICA.** Feb 1902, *Donnell-Smith 6824* (B, iso); 20 Feb 1989, *Russell 849* (CR); 18 Feb 1990, *Grant 799* (CR, SEL); 17 Oct 1996, *Moraga 769* (CR, INB); 1 Mar 2007, *Rodríguez 10930* (INB).

**Life form.** Epiphyte (occasionally terrestrial).

***Guzmania glomerata*** Mez & Wercklé, Repert. Spec. Nov. Regni. Veg. 14: 256 (1916).

**Distribution.** Nicaragua, Costa Rica, Panama, Colombia, and Ecuador.

**Representative Collections—COSTA RICA.** *Wercklé s.n.* (B, holo); 20 Aug 1989, *Haber 9465* (CR, INB); 20 Aug 1993, *Palaci 1216* (INB); 27 Jul 1996, *Hammel 20312* (INB); 1 Dec 1999, *Rodríguez 5528* (INB).

**Life form.** Epiphyte.

***^1^*Guzmania herrerae*** H. Luther & W. J. Kress, Brittonia 48: 91 (1996).

**Distribution.** Costa Rica.

**Representative Collections—COSTA RICA.** 16 Nov 1987, *Herrera 1322* (CR, holo; MO, iso); 8 Jul 1988, *Herrera 2014* (INB); 12 Oct 1995, *Cascante 787* (CR); 20 Sep 2003, *Kriebel 3838* (INB); 27 Oct 2007, *Solano 4755* (INB).

**Life form.** Epiphyte.

***Guzmania lingulata*** (L.) Mez, Monogr. Phan. 9: 899 (1896).

**Distribution.** Mexico, Guatemala, Belize, Honduras, Nicaragua, Costa Rica, Panama, Colombia, Ecuador, Peru, Bolivia, Brazil, Suriname, Guianas, Venezuela, Trinidad and Tobago, and Dominican Republic.

**Representative Collections—COSTA RICA.** 20 Aug 1964, *Lent 233* (CR); 21 Sep 1986, *Davidse 31521* (CR); 31 May 1990, *Herrera 3938* (INB, SEL); 28 Jun 1994, *Kress 94-4055* (CR, USJ); 11 Jun 2003, *Alfaro 4488* (INB).

**Life form.** Epiphyte.

***Guzmania monostachia*** (L.) Rusby ex Mez, Monogr. Phan. 9: 905 (1896).

**Distribution.** Honduras, Nicaragua, Costa Rica, Panama, Colombia, Ecuador, Peru, Bolivia, Venezuela, Trinidad and Tobago, Puerto Rico, Dominican Republic, Haiti, Jamaica, and United States of America.

**Representative Collections—COSTA RICA.** 1 May 1913, *Tonduz s.n.* (CR); 1 Aug 1949, *Holm 782* (CR); 10 Aug 1967, *Taylor 4276* (NY); 7 Jan 1992, *Grant 92-1761* (CR, INB, SEL); 28 May 2003, *Clark 268* (SEL).

**Life form.** Epiphyte.

***Guzmania musaica*** (Linden & André) Mez, Monogr. Phan. 9: 898 (1896).

**Distribution.** Costa Rica, Panama, Colombia, Ecuador, and Venezuela.

**Representative Collections—COSTA RICA.** 7 Jul 1989, *Hammel 17600* (CR, INB); 9 Jul 1989, *Chacón 138* (INB); 25 Jul 1989, *Chacón 278* (INB).

**Life form.** Epiphyte.

***Guzmania nicaraguensis*** Mez & C. F. Baker ex Mez, Bull. Torrey Bot. Club 30: 436 (1903).

**Distribution.** Mexico, Guatemala, Belize, Honduras, Nicaragua, Costa Rica, Panama, and Ecuador.

**Representative Collections—COSTA RICA.** 20 Jan 1939, *Smith 1529* (NY); 7 Feb 1940, *Brenes 23012* (NY); 17 Feb 1992, *Ingram 1350* (SEL); 26 Apr 2001, *Mora 1994* (NY; CR; INB; MO, n.v.); 22 Aug 2007, *Morales 15497* (INB).

**Life form.** Epiphyte.

***Guzmania obtusiloba*** L. B. Sm., Contr. Gray Herb. 104: 74 (1934).

**Distribution.** Costa Rica, Panama, and Colombia.

**Representative Collections—COSTA RICA.** 15 Aug 1933, *Valerio 702* (CR); 28 Nov 1971, *Lent 2254* (CR, n.v.; NY; PMA); 20 Feb 1990, *Grant 814* (CR, SEL); 25 Aug 2000, *Homeier & Vora 496* (USJ); 27 Oct 2007, *Monro 5847* (INB).

**Life form.** Epiphyte (rarely terrestrial).

***Guzmania patula*** Mez & Wercklé, Repert. Sp. Nov. Regni. Veg. 14: 255 (1916).

**Distribution.** Costa Rica, Panama, Colombia, Ecuador, and Venezuela.

**Representative Collections—COSTA RICA.** *Wercklé s.n.* (B, holo; US, iso, n.v.); 23 Oct 1964, *Jiménez 2490* (SCZ); 1 Jul 1976, *Ocampo 1356* (CR); 8 Jul 1997, *Estrada 946* (CR); 8 Aug 2007, *Soto 1644* (INB, NY).

**Life form.** Epiphyte.

**^2^*Guzmania plicatifolia*** L. B. Sm., Contr. Gray Herb. 102 68: 146 (1933).

**Distribution.** Costa Rica and Panama.

**Representative Collections—COSTA RICA.** 10 Jun 1925, *Brenes 7473* (CR); 22 May 1977, *Dwyer 1365* (CR); 17 Feb 1992, *Ingram 1340* (INB, SEL); 21 Jul 1994, *Lépiz 501* (CR, INB); 8 Jun 2000, *Chaves 568* (INB).

**Life form.** Epiphyte and terrestrial.

***Guzmania polycephala*** Mez & Wercklé ex Mez, Repert. Spec. Nov. Regni. Veg. 14: 254 (1916).

**Distribution.** Costa Rica, Panama, and Colombia.

**Representative Collections—COSTA RICA.** *Wercklé s.n.* (B, holo); Apr 1989, *Hall s.n.* (SEL); 3 Jun 2001, *Morales 8176* (CR, INB); 3 Aug 2006, *Cascante 1597* (CR); 6 Mar 2007, *Solano 4204* (INB).

**Life form.** Epiphyte.

***Guzmania sanguinea*** (André) André ex Mez, Monogr. Phan. 9: 901 (1896).

**Distribution.** Costa Rica, Panama, Colombia, and Ecuador.

**Representative Collections—COSTA RICA.** *Wercklé 84* (B, lecto); 1 Mar 1970, *Gómez 3272* (CR); 8 Mar 1994, *Morales 2460* (INB, SEL); 22 Jun 1997, *Rojas 3656* (CR, INB); 30 Aug 2001, *Cascante 1545* (CR).

**Life form.** Epiphyte.

**^2^*Guzmania scandens*** H. Luther & W. J. Kress, Brittonia 48: 93 (1996).

**Distribution.** Costa Rica and Panama.

**Representative Collections—COSTA RICA.** 15 Aug 1933, *Valerio 721* (CR); 10 Jun 1968, *Burger 5743* (CR); 28 Jul 1990, *Luther 2816* (CR; SEL, holo); 2 Jun 1994, *Lépiz 321* (CR, INB); 28 Oct 2007, *Santamaría 6684* (INB).

**Life form.** Epiphyte (rarely terrestrial or saxicolous).

***Guzmania scherzeriana*** Mez, Monogr. Phan. 9: 949 (1896).

**Distribution.** Guatemala, Belize, Honduras, Nicaragua, Costa Rica, Panama, Colombia, and Ecuador.

**Representative Collections—COSTA RICA.** 12-15 Aug 1971, *Burger & Burger 8084* (NY, PMA); 20 Aug 1989, *Haber 9470* (CR, INB); 1 Aug 1994, *Alverson 3209* (CR, USJ); 28 May 2005, *Solano 2458* (INB); 2 Jul 2008, *Vargas 3399* (INB).

**Life form.** Epiphyte.

***^1^*Guzmania skotakii*** H. Luther, Selbyana 12: 68 (1991).

**Distribution.** Costa Rica.

**Representative Collections—COSTA RICA.** 23 Jul 1990, *Luther 2810* (CR; SEL, holo); 28 Jul 1990, *Luther 2817* (SEL); 17 Jan 1991, *Grant 1510* (INB, SEL); 22 Jul 1994, *Morales 3073* (INB).

**Life form.** Epiphyte.

**^1^*Guzmania spectabilis*** (Mez & Wercklé) Utley, Phytologia 40(1): 55 (1978).

**Distribution.** Costa Rica and Ecuador.

**Representative Collections—COSTA RICA.** *Wercklé s.n.* (B, holo); 6 Mar 1992, *Bittner 1438* (INB); 22 Jun 1998, *Valverde 1005* (CR); 23 Sep 1998, *Estrada 1745* (CR); 25 Feb 2001, *Morales 7618* (INB).

**Life form.** Epiphyte.

***Guzmania sprucei*** (André) L. B. Sm, Contr. Gray Herb. 104: 75 (1934).

**Distribution.** Costa Rica, Panama, and Colombia.

**Representative Collections—COSTA RICA.** 19 Apr 1988, *Kress 2406* (SEL); 10 Oct 1989, *Vargas 225* (CR, INB); 8 Sep 1990, *Solomon 19255* (CR); 24 Sep 1994, *Gallardo 301* (CR, INB); 2 Nov 2007, *Rodríguez 11675* (INB).

**Life form.** Epiphyte.

**^2^*Guzmania stenostachya*** L. B. Sm., Contr. Gray Herb. 117: 9 (1937).

**Distribution.** Costa Rica and Panama.

**Representative Collections—COSTA RICA.** 4 May 1975, *Utley 2371* (CR); 14 May 1983, *Liesner 15559* (CR, SEL); 12 Jul 1992, *Kress 3487* (INB, SEL); 28 May 2005, *Morales 13106* (INB); 8 Aug 2007, *Soto 1647* (INB, NY).

**Life form.** Epiphyte (occasionally terrestrial).

***Guzmania subcorymbosa*** L. B. Sm., Contr. Gray Herb. 117: 10 (1937).

**Distribution.** Costa Rica, Panama, and Colombia.

**Representative Collections—COSTA RICA.** 15 Oct 1987, *Herrera 874* (CR, INB); 18 Feb 1990, *Grant 800* (INB, SEL); 14 Apr 1995, *Moraga 126* (CR, INB); 7 Dec 1996, *Moraga 843* (INB); 8 Jan 2000, *Morales 7234* (INB).

**Life form.** Epiphyte.

***Guzmania zahnii*** (Hooker f.) Mez, Monogr. Phan. 9: 940 (1896).

**Distribution.** Nicaragua, Costa Rica, and Panama.

**Representative Collections—COSTA RICA.** 5 Aug 1980, *Meerow 2020* (CR, SEL); Aug 1991, *Luther s.n.* (SEL); 30 Aug 2001, *Cascante 1546* (CR); 7 Apr 2005, *Morales 12670* (INB); 19 Jun 2007, *de Melo & Moran 8032* (NY).

**Life form.** Epiphyte.

***MEZOBROMELIA***

***Mezobromelia pleiosticha*** (Griseb.) Utley & H. Luther, Ann. Missouri Bot. Gard. 78: 270 (1991).

**Distribution.** Costa Rica, Panama, Colombia, Ecuador, Peru, Bolivia, and Venezuela.

**Representative Collections—COSTA RICA.** Feb 1969, *Lankester s.n.* (SEL); 22 Jul 1994, *Morales 3072* (CR, INB); 21 Jun 1995, *Herrera 8020* (CR); 21 Aug 2002, *Estrada 3328* (CR); 17 Jun 2005, *Morales 13196* (INB).

**Life form.** Epiphyte.

***PEPINIA***

***^1^*Pepinia beachiae*** (Utley & Burt-Utley) H. Luther, Phytologia 74: 449 (1993).

**Syn.:** *Pitcairnia beachiae* Utley & Burt-Utley, Ann. Missouri Bot. Gard. 78: 266 (1991).

**Distribution.** Costa Rica.

**Representative Collections—COSTA RICA.** 24 Jul 1974, *Beach 74-25* (SEL); 27 Jul 1990, *Luther s.n.* (SEL); 21 May 1992, *Luther s.n.* (SEL).

**Life form.** Epiphyte and terrestrial.

***PITCAIRNIA***

***Pitcairnia arcuata*** (André) André, Rev. Hort. 60: 565 (1888).

**Distribution.** Costa Rica, Panama, and Colombia.

**Representative Collections—COSTA RICA.** 16 Apr 1983, *Liesner 14378* (CR); 22 Jun 1990, *Luther 2800* (MO, SEL); 5 Mar 1994, *Boyle 2877* (CR, INB, SEL); 17 Nov 1999, *Acosta 132* (CR, INB); 14 May 2006, *Cascante 1565* (CR).

**Life form.** Epiphyte and terrestrial.

***Pitcairnia atrorubens*** (Beer) Baker, J. Bot. 19: 307 (1881).

**Distribution.** Guatemala, Honduras, Costa Rica, Panama, and Colombia.

**Representative Collections—COSTA RICA.** 9 Aug 1898, *Tonduz 12532* (CR); 19–20 Dec 1966, *Burger & Ramirez 4011* (CR, NY); 22 Feb 1990, *Grant 846* (CR, SEL); 6 Sep 1995, *Jiménez 1909* (CR, INB); 27 Jul 2007, *Bridgewater 4221* (INB).

**Life form.** Epiphyte and terrestrial.

***Pitcairnia brittoniana*** Mez, Monogr. Phan. 9: 451 (1896).

**Distribution.** Belize, Nicaragua, Costa Rica, Panama, Colombia, Ecuador, and Venezuela.

**Representative Collections—COSTA RICA.** *Wercklé 108* (B, holo); 7 Jul 1964, *Jiménez 2014* (CR, NY); 14 May 1983, *Liesner 15466* (CR, SEL); 27 Jul 2004, *Solano 1205* (INB); 5 Mar 2008, *Morales 15976* (INB).

**Life form.** Epiphyte and terrestrial.

***^1^*Pitcairnia calcicola*** J. R. Grant & J. F. Morales, Novon 6: 366 (1996).

**Distribution.** Costa Rica.

**Representative Collections—COSTA RICA.** 17 Jul 1992, *Grant 92-2008* (CR, SEL); 3 May 1995, *Espinoza 1276* (CR, INB); 27 Jan 2000, *Rodríguez 5640* (INB); 17 Nov 2004, *Morales 11589* (INB); 20 Nov 2005, *Hammel 23887* (INB).

**Life form.** Terrestrial and saxicolous.

***^1^*Pitcairnia funkiae*** M. A. Spencer & L. B. Sm., J. Bromeliad Soc. 41: 214 (1991).

**Distribution.** Costa Rica.

**Representative Collections—COSTA RICA.** 16 Jul 1992, *Grant 1982* (CR, SEL); 27 Jul 1991, *Rivera 1478* (INB); 15 Jan 1992, *Grant 1887* (INB); 26 May 2003, *Holst 8553* (SEL); 30 Apr 2004, *Rodríguez 8775* (INB).

**Life form.** Epiphyte and terrestrial.

***Pitcairnia guzmanioides*** L. B. Sm., Contr. U.S. Natl. Herb. 29: 306 (1949).

**Distribution.** Costa Rica, Colombia, Ecuador, and Peru.

**Representative Collections—COSTA RICA.** 22 May 1994, *Morales 3365* (INB); 22 Jun 1994, *Morales 3343* (INB); 21 Jul 1994, *Lépiz 509* (INB); 17 Jun 2005, *Morales 13207* (INB); 8 Jul 2005, *Solano 2592* (INB).

**Life form.** Terrestrial (rarely epiphyte).

**^1,2^*Pitcairnia halophila*** L. B. Sm., Phytologia 10: 32 (1964).

**Distribution.** Costa Rica and Panama.

**Representative Collections—COSTA RICA.** 24 Jul 1985, *Soto 2348* (CR); 21 Jan 1987, *Grayum 7979* (CR, INB); 18 Dec 1989, *Merz 565* (CR); 21 Dec 1998, *Morales 6879* (CR, INB); 28 Nov 2004, *Hammel 23446* (INB).

**Life form.** Terrestrial and saxicolous.

***Pitcairnia heterophylla*** (Lindl.) Beer, Fam. Bromel.: 68 (1856).

**Distribution.** Mexico, Guatemala, Belize, El Salvador, Honduras, Nicaragua, Costa Rica, Panama, Colombia, Ecuador, Peru, and Venezuela.

**Representative Collections—COSTA RICA.** Dec 1899, *Tonduz 13645* (B); Dec 1936, *Skutch 3105* (NY); 4 Oct 1987, *Herrera 769* (CR, INB); 11 Feb 1995, *Haber 11900* (CR, INB); 19 Jun 2003, *Moran & Noguera 6446* (NY).

**Life form.** Epiphyte, terrestrial, and saxicolous.

***Pitcairnia kalbreyeri*** Baker, J. Bot. 19: 273 (1881).

**Distribution.** Costa Rica, Panama, Colombia, Ecuador, and Peru.

**Representative Collections—COSTA RICA.** 14 Apr 1975, *Utley 2116* (CR); 11 Feb 1986, *Gómez-Laurito 11050* (CR); 14 Mar 1990, *Haber 9807* (CR, INB); 21 May 2005, *Morales 13014* (INB); 18 Feb 2007, *Santamaría 5703* (INB).

**Life form.** Terrestrial (rarely epiphyte).

**^2^*Pitcairnia lymansmithiana*** H. Luther, J. Bromeliad Soc. 37: 212 (1987).

**Distribution.** Costa Rica and Panama.

**Representative Collections—COSTA RICA.** Jul 1992, *Hidalgo s.n.* (SEL); 2 Jun 1994, *Lépiz 325* (INB); 22 Jul 1994, *Lépiz 516* (INB).

**Life form.** Terrestrial.

***Pitcairnia maidifolia*** (C. Morren) Decne., Fl. Serres Jard. Eur. 9: 151 (1854).

**Distribution.** Honduras, Nicaragua, Costa Rica, Panama, Colombia, Ecuador, Peru, and Venezuela.

**Representative Collections—COSTA RICA.** 14 Nov 1985, *Gómez-Laurito 10692* (CR); 5 Dec 1990, *Herrera 4708* (CR, INB); 22 Feb 1991, *Till 7072* (CR); 25 Nov 1994, *Jiménez 1669* (CR, INB, MO, NY); 9 Dec 2004, *Solano 1610* (INB).

**Life form.** Epiphyte, terrestrial, and saxicolous.

***Pitcairnia megasepala*** Baker, J. Bot. 19: 229 (1881).

**Distribution.** Costa Rica, Panama, and Colombia.

**Representative Collections—COSTA RICA.** Mar 1892, *Tonduz 6868* (B, iso; NY); 1 Nov 1978, *Ocampo 2264* (CR); 5 Jan 1992, *Grant 1739* (CR, INB, SEL); 14 Nov 2000, *Acosta 2931* (INB); 20 Oct 2005, *Morales 13415* (INB).

**Life form.** Epiphyte and terrestrial (rarely saxicolous).

***^1^*Pitcairnia membranifolia*** Baker, Handb. Bromel.: 109 (1889).

**Distribution.** Costa Rica.

**Representative Collections—COSTA RICA.** 23 May 1981, *Gómez-Laurito 6721* (CR); 23 May 1981, *Gómez-Laurito 6722* (INB); May 1986, *Skotak s.n.* (SEL); 22 Aug 1998, *Morales 6470* (INB); 16 Oct 2000, *Morales 7384* (INB).

**Life form.** Terrestrial, saxicolous, very rarely epiphytic.

***Pitcairnia quesnelioides*** L. B. Sm., Contr. U. S. Natl. Herb. 29: 313 (1949).

**Distribution.** Costa Rica and Colombia.

**Representative Collections—COSTA RICA.** 3 Aug 1990, *Herrera 4113* (INB); 24 Apr 2004, *Morales 10447* (INB); 16 Aug 2005, *Morales 13266* (INB).

**Life form.** Terrestrial.

***Pitcairnia saxicola*** L. B. Sm., Contr. Gray Herb. 117: 29 (1937).

**Distribution.** Mexico, Honduras, Costa Rica, and Panama.

**Representative Collections—COSTA RICA.** 6 Jan 1994, *Grant 94-2301* (CR); 2 Jan 1997, *Morales 5980* (INB); 18 Oct 2001, *Lobo 422* (CR); 12 Dec 2001, *Bustamante 227* (INB).

**Life form.** Saxicolous (rarely epiphyte).

**^2^*Pitcairnia valerioi*** Standl., J. Wash. Acad. Sci. 17: 246 (1927).

**Distribution.** Costa Rica and Panama.

**Representative Collections—COSTA RICA.** 29 Jun 1925, *Brenes 4261* (CR); 3 Feb 1986, *Grayum 6333* (CR); 19 Apr 1988, *Kress 88-2415* (SEL); 20 Apr 1988, *Hammel 16702* (CR, INB); 26 Oct 2007, *Solano 4733* (INB).

**Life form.** Terrestrial and epiphyte (rarelly saxicolous).

***Pitcairnia wendlandii*** Baker, J. Bot. 19: 306 (1881).

**Distribution.** Mexico, Guatemala, Belize, Costa Rica, and Panama.

**Representative Collections—COSTA RICA.** 20-22 Dec 1969, *Burger 6985* (NY); 1 Jul 1974, *Ocampo 688* (CR); 31 May 1991, *Bittner 1043* (CR, INB); 5 Sep 1993, *Herrera 6509* (CR); 2 Mar 2000, *Acosta 513* (INB).

**Life form.** Terrestrial and epiphyte.

***PUYA***

***^1^*Puya dasylirioides*** Standl., J. Wash. Acad. Sci. 17: 159 (1927).

**Distribution.** Costa Rica.

**Representative Collections—COSTA RICA.** 30 Jul 1962, *Lee 760* (CR); 23 Jan 1965, *Lems 5186* (NY); 8 Jul 1986, *Atwood 27* (SEL); 8 Apr 1995, *Aguilar 4006* (CR, INB); 15 Jun 2005, *Morales 13139* (INB).

**Life form.** Terrestrial.

***Puya floccosa*** (Linden) E. Morren ex Mez, Monogr. Phan. 9: 478 (1896).

**Distribution.** Costa Rica, Colombia, and Venezuela.

**Representative Collections—COSTA RICA.** 12 Mar 1993, *Fernández 612* (INB); 9 Sep 1995, *Jiménez 1998* (INB); 22 Nov 2005, *Solano 2877* (INB); 30 May 2006, *González 4184* (CR); 15 Jun 2006, *Santamaría 4569* (INB).

**Life form.** Terrestrial (rarely saxicolous).

***RACINAEA***

***Racinaea adpressa*** (André) J. R. Grant, Novon 4(4): 362 (1994).

**Distribution.** Costa Rica, Panama, Ecuador, Peru, Bolivia, and Guianas.

**Representative Collections—COSTA RICA.** 3 Jan 1990, *Bello 1656* (INB); 10 Jan 1992, *Bello 4310* (CR, INB); 25 Mar 1993, *Fernández 830* (INB); 3 Apr 1994, *Morales 2626* (INB, SEL); 29 Jul 1996, *Gamboa 519* (INB).

**Life form.** Epiphyte.

***Racinaea contorta*** (Mez) M. A. Spencer & L. B. Sm., Phytologia 74: 153 (1993).

**Distribution.** Nicaragua, Costa Rica, Panama, Colombia, and Ecuador.

**Representative Collections—COSTA RICA.** 26 May 1973, *Ocampo 405* (CR); 3 May 1984, *Gómez 21195* (INB, SEL); Jul 1985, *Cathcart s.n.* (SEL); 10 Apr 1991, *Smith 10853* (CR); 23 Aug 2000, *Moraga 1121* (INB).

**Life form.** Epiphyte.

***Racinaea rothschuhiana*** (Mez) M. A. Spencer & L. B. Sm., Phytologia 74: 157 (1993).

**Distribution.** Mexico, Guatemala, Honduras, Nicaragua, and Costa Rica.

**Representative Collections—COSTA RICA.** Jul 1985, *Cathcart s.n.* (SEL); 6 Apr 1994, *Morales 2655* (INB); 20 May 1996, *Morales 5365* (INB); 16 Feb 2005, *Morales 11993* (INB); 11 May 2005, *Morales 12927* (INB).

**Life form.** Epiphyte.

***Racinaea schumanniana*** (Wittm.) J. R. Grant, Novon 4: 363 (1994).

**Distribution.** Costa Rica, Panama, Colombia, Ecuador, Peru, Bolivia, and Venezuela.

**Representative Collections—COSTA RICA.** 11 Jan 1896, *Tonduz 12670* (CR); 22 Jul 1990, *Luther 2804* (CR, SEL); 2 Dec 1993, *Lépiz 640* (INB, MO, NY); 19 Jul 2000, *Rodríguez 5986* (CR, INB); 10 May 2005, *Morales 12878* (INB).

**Life form.** Epiphyte.

***Racinaea spiculosa*** (Griseb.) M. A. Spencer & L. B. Sm., Phytologia 74: 157 (1993).

**Distribution.** Nicaragua, Costa Rica, Panama, Colombia, Ecuador, Peru, Bolivia, Brazil, Suriname, Guianas, Venezuela, and Dominican Republic.

**Representative Collections—COSTA RICA.** 1 Jan 1901, *Wercklé 16175* (CR); Jan 1964, *Lankester s.n.* (SEL); 14 Jan 1992, *Grant 92-1867* (CR); 11 Mar 2001, *Cascante 1501* (CR); 20 Jun 2004, *Morales 10808* (INB).

**Life form.** Epiphyte.

***RONNBERGIA***

***Ronnbergia hathewayi*** L. B. Sm., Phytologia 15: 196 (1967).

**Distribution.** Costa Rica, Panama, and Colombia.

**Representative Collections—COSTA RICA.** 13 Feb 1984, *Gómez. 21065* (CR); 19 Apr 1988, *Kress 88-2405* (INB, SEL); 12 Jul 1995, *Morales 4858* (INB); 6 Mar 2001, *Mora 1838* (CR, INB); 28 Oct 2007, *Monro 5861* (INB).

**Life form.** Epiphyte and terrestrial.

***TILLANDSIA***

***^1^*Tillandsia abdita*** L. B. Sm., Phytologia 8: 10 (1961).

**Distribution.** Costa Rica.

**Representative Collections—COSTA RICA.** 31 Jul 1935, *Solís 314* (CR, NY); 25 Jun 1995, *Morales 4614* (INB); 16 Apr 1994, *Morales 2692* (INB); 22 Jul 2000, *Morales 7332* (INB).

**Life form.** Epiphyte.

***Tillandsia anceps*** Lodd., Bot. Cab. 8: t. 771 (1823).

**Distribution.** Guatemala, Belize, Nicaragua, Costa Rica, Panama, Colombia, Ecuador, Brazil, Guianas, and Venezuela.

**Representative Collections—COSTA RICA.** 1 Apr 1913, *Tonduz s.n.* (CR); 29 Sep 1985, *Atwood 8516* (SEL); 14 Jul 1989, *Herrera 3289* (CR, INB); 30 Jul 2001, *Acosta 3129* (CR, INB); 28 May 2005, *Morales 13112* (INB).

**Life form.** Epiphyte.

***Tillandsia balbisiana*** Schult. f., Syst. Veg. 7(2): 1212 (1830).

**Distribution.** Mexico, Guatemala, Belize, El Salvador, Honduras, Nicaragua, Costa Rica, Panama, Colombia, Ecuador, Venezuela, Dominican Republic, Jamaica, Cuba, and United States of America.

**Representative Collections—COSTA RICA.** 1 Aug 1949, *Holm 781* (CR); 8 Jul 1987, *Zamora 1348* (CR, INB); 6 Jan 1992, *Grant 1756* (CR, INB, SEL); 23 Aug 1995, *Sanders 17699* (CR, SEL); 26 Sep 2000, *Acosta 2743* (CR, INB).

**Life form.** Epiphyte.

***Tillandsia biflora*** Ruiz & Pav., Fl. Peruv. 3: 41 (1802).

**Distribution.** Honduras, Nicaragua, Costa Rica, Panama, Colombia, Ecuador, Peru, Bolivia, and Venezuela.

**Representative Collections—COSTA RICA.** 18 Aug 1995, *Picado 286* (CR, INB); 18 Sep 1996, *Morales 5804* (CR, INB); 10 Jul 1998, *Boyle 5433* (CR); 20 Sep 2003, *Alfaro 5207* (INB); 25 Jun 2006, *Morales 13988* (INB).

**Life form.** Epiphyte.

***Tillandsia brachycaulos*** Schltdl., Linnaea 18: 422 (1845).

**Distribution.** Mexico, Guatemala, Belize, El Salvador, Honduras, Nicaragua, Costa Rica, Panama, and Venezuela.

**Representative Collections—COSTA RICA.** 7 Jan 1890, *Pittier 2927* (CR); 30 May 1932, *Brenes 15614* (NY); 1 Jul 1964, *Gilmartin 931* (SEL); 19 Aug 2000, *Acosta 2504* (CR, INB); 15 Dec 2004, *Hammel 23488* (INB).

**Life form.** Epiphyte (rarely saxicolous).

***Tillandsia bulbosa*** Hook., Exot. Fl.: t. 173 (1826).

**Distribution.** Mexico, Guatemala, Belize, Honduras, Nicaragua, Costa Rica, Panama, Colombia, Ecuador, Guianas, Venezuela, Dominican Republic, and Jamaica.

**Representative Collections—COSTA RICA.** 22 Jun 1966, *Jiménez 4031* (CR); 9 Apr 1983, *Liesner 14151* (CR); 27 Mar 1995, *Herrera 7593* (CR); 29 May 2003, *Clark 301* (SEL); 7 Apr 2005, *Solano 2094* (INB).

**Life form.** Epiphyte.

***Tillandsia butzii*** Mez, Pflanzenr., IV, 32: 636 (1935).

**Distribution.** Mexico, Guatemala, Belize, El Salvador, Honduras, Nicaragua, Costa Rica, and Panama.

**Representative Collections—COSTA RICA.** 5 Jan 1890, *Pittier 2615* (CR); 12 Dec 1976, *Lent 3957* (NY); 16 Jan 1991, *Grant 1467* (CR, SEL); 27 Jan 1998, *Rodríguez 2938* (INB, MO, NY); 6 Aug 2008, *Cascante 2006* (CR).

**Life form.** Epiphyte.

***Tillandsia caput-medusae*** E. Morren, Ann. Bot. Hort. 30: 90 (1880).

**Distribution.** Mexico, Guatemala, Belize, El Salvador, Honduras, Nicaragua, Costa Rica, and Panama.

**Representative Collections—COSTA RICA.** 21 Dec 1974, *Taylor 17383* (NY); 24 Apr 1988, *Kress 2446* (SEL); 7 Jan 1990, *Merz 570* (CR); 26 Jul 1994, *Estrada 103* (CR, INB); 23 Apr 2005, *Morales 12807* (INB).

**Life form.** Epiphyte.

***^1^*Tillandsia cauliflora*** Mez & Wercklé, Bull. Herb. Boissier, II, 5: 100 (1905).

**Distribution.** Costa Rica.

**Representative Collections—COSTA RICA.** *Wercklé 68* (B, holo; NY, photo); Feb 1984, *Waggoner s.n.* (SEL); Jun 1990, *Tristram s.n.* (SEL); 20 Jul 1995, *Morales 4597* (CR, INB, MO).

**Life form.** Epiphyte.

***Tillandsia complanata*** Benth., Bot. Voy. Sulphur: 173 (1846).

**Distribution.** Costa Rica, Panama, Colombia, Ecuador, Peru, Bolivia, Venezuela, and Jamaica.

**Representative Collections—COSTA RICA.** 10 Jul 1962, *Lee 671* (CR); 19-20 Dec 1966, *Burger 4023* (CR, NY); 27 Feb 1990, *Grant 898* (SEL); 6 Apr 1994, *Morales 2639* (CR, INB); 19 Jul 2007, *Cascante 1788* (CR).

**Life form.** Epiphyte.

**^1,2^*Tillandsia dexteri*** H. Luther, Selbyana 11: 54 (1989).

**Distribution.** Costa Rica and Panama.

**Representative Collections—COSTA RICA.** 24 Feb 1988, *Dexter s.n.* (SEL, holo); 28 Jul 1988, *Skotak s.n.* (SEL); 30 Sep 1995, *Tust s.n.* (SEL).

**Life form.** Epiphyte.

***Tillandsia elongata*** Kunth, Nov. Gen. Sp. 1: 293 (1816).

**Distribution.** Nicaragua, Costa Rica, Panama, and Colombia.

**Representative Collections—COSTA RICA.** 22 Aug 2005, Morales 13326 (INB).

**Life form.** Epiphyte.

***Tillandsia excelsa*** Griseb., Fl. Brit. W. I.: 597 (1864).

**Distribution.** Mexico, Guatemala, Belize, Honduras, Nicaragua, Costa Rica, Panama, and Jamaica.

**Representative Collections—COSTA RICA.** Nov 1897, *Tonduz 11375* (B, lecto); 9 Aug 1967, *Taylor 4195* (NY); 3 Sep 1989, *Hammel 17683* (CR, INB); 22 Jul 1990, *Luther 2807* (SEL); 22 Feb 2008, *Rodríguez 11835* (INB).

**Life form.** Epiphyte.

***Tillandsia fasciculata*** Sw., Prod. Veg. Ind. Occ.: 56 (1788).

**Distribution.** Mexico, Guatemala, Belize, El Salvador, Honduras, Nicaragua, Costa Rica, Panama, Colombia, Brazil, Guianas, Venezuela, Virgin Islands, Puerto Rico, Dominican Republic, Haiti, Jamaica, and United States of America.

**Representative Collections—COSTA RICA.** 5 Jan 1890, *Pittier 2616* (CR); 1 Aug 1949, *Holm 772* (CR); 18 Feb 1991, *Till 7026* (CR); 11 Oct 1995, *Jiménez 2044* (CR, INB); 5 Feb 2008, *Cascante 1840* (CR).

**Life form.** Epiphyte.

***Tillandsia festucoides*** Brongn. ex Mez, Monogr. Phan. 9: 678 (1896).

**Distribution.** Mexico, Guatemala, Belize, Honduras, Nicaragua, Costa Rica, Panama, Dominican Republic, and Jamaica.

**Representative Collections—COSTA RICA.** 24-26 Apr 1906, *Maxon 166* (NY); 20-22 Dec 1969, *Burger 6875* (CR, PMA); 14 Apr 1994, *Gallardo 138* (CR, INB, NY); 18 Jan 2002, *Ferrufino & González 180* (USJ); 23 Apr 2006, *Morales 13858* (INB).

**Life form.** Epiphyte.

***Tillandsia filifolia*** Schltdl. & Cham., Linnaea 6: 53 (1831).

**Distribution.** Mexico, Belize, Honduras, Nicaragua, and Costa Rica.

**Representative Collections—COSTA RICA.** 24 Jul 1972, *Ocampo 716* (CR); 6 Jul 1983, *Gómez-Laurito 9556* (CR); 5 May 1983, *Liesner 15289* (INB); 14 Apr 1994, *Gallardo 128* (INB); 23 Apr 2006, *Morales 13857* (INB).

**Life form.** Epiphyte.

***Tillandsia flexuosa*** Sw., Prod. Veg. Ind. Occ.: 56 (1788).

**Distribution.** Costa Rica, Panama, Colombia, Bolivia, Brazil, Venezuela, Trinidad and Tobago, Dominican Republic, Haiti, Cuba, and United States of America.

**Representative Collections—COSTA RICA.** 27 Jul 1990, *Luther s.n.* (SEL); 20 Oct 1993, *Quesada 769* (INB); 22 Oct 1993, *Morales 1932.1* (INB); 14 Sep 1999, *Takizawa 99-f* (SEL).

**Life form.** Epiphyte.

***Tillandsia guatemalensis*** L. B. Sm., Contr. U. S. Natl. Herb. 29: 281 (1949).

**Distribution.** Mexico, Guatemala, El Salvador, Honduras, Nicaragua, Costa Rica, and Panama.

**Representative Collections—COSTA RICA.** 16 May 2000, *Alfaro 3159* (INB).

**Life form.** Epiphyte.

***Tillandsia ionantha*** Planch., Fl. Serres Jard. Eur. 10: 101 (1855).

**Distribution.** Mexico, Guatemala, El Salvador, Honduras, Nicaragua, Costa Rica, Panama, and Peru.

**Representative Collections—COSTA RICA.** 22 Nov 1973, *Solomon s.n.* (CR); 17 Dec 1986, *Soto 3300* (CR); 18 Mar 1992, *Skotak s.n.* (SEL); 13 Sep 1995, *Sanders 17882* (SEL); 25 Apr 2001, *Acosta 3052* (INB).

**Life form.** Epiphyte.

***Tillandsia juncea*** Poir., Encycl., Suppl. 5: 309 (1817).

**Distribution.** Mexico, Guatemala, Belize, El Salvador, Honduras, Nicaragua, Costa Rica, Panama, Colombia, Ecuador, Peru, Bolivia, Venezuela, Dominican Republic, and Haiti.

**Representative Collections—COSTA RICA.** 5 Jan 1890, *Pittier 2617* (CR); 18 Feb 1991, *Till 7030* (CR); 10 Jan 1992, *Grant 92-1828* (CR, INB, NY, SEL); 10 May 2003, *Alfaro 4374* (CR, INB); 6 Aug 2008, *Cascante 2008* (CR).

**Life form.** Epiphyte.

***Tillandsia lampropoda*** L. B. Sm., Publ. Field Mus. Nat. Hist., Bot. Ser. 17: 320 (1938).

**Distribution.** Mexico, Guatemala, El Salvador, Honduras, Nicaragua, and Costa Rica.

**Representative Collections—COSTA RICA.** 22 Jan 1994, *Morales 2298* (INB); 23 Dec 1997, *Morales 6341* (INB); 11 May 2005, *Morales 12926* (INB).

**Life form.** Epiphyte.

***Tillandsia leiboldiana*** Schltdl., Linnaea 18: 414 (1844).

**Distribution.** Mexico, Belize, Honduras, Nicaragua, Costa Rica, and Panama.

**Representative Collections—COSTA RICA.** 1 Jan 1901, *Wercklé 16177* (B, CR); 25 Aug 1975, *Utley & Utley 2981* (NY); 19 May 1989, *Kress 89-2813* (CR, INB, SEL); 18 Nov 1992, *Ingram 1742* (SEL); 20 Feb 2008, *Santamaría 7016* (INB).

**Life form.** Epiphyte.

***Tillandsia longifolia*** Baker, Handb. Bromel.: 185 (1889).

**Distribution.** Costa Rica, Panama, Peru, Bolivia, and Venezuela.

**Representative Collections—COSTA RICA.** 5 Apr 1974, *Utley 791* (CR); 19 Aug 1983, *Herrera 21625* (CR); 23 Jan 1996, *Morales 5106* (CR, INB); 13 Nov 2000, *Acosta 2912* (INB); 24 Feb 2008, *Santamaría 7140* (INB).

**Life form.** Epiphyte.

***Tillandsia makoyana*** Baker, Handb. Bromel.: 189 (1889).

**Distribution.** Mexico, Guatemala, El Salvador, Honduras, Nicaragua, and Costa Rica.

**Representative Collections—COSTA RICA.** 15 Jul 1992, *Grant 1972* (INB, MO, SEL); 9 Nov 1996, *Oconnor 70* (INB); 16 Mar 2002, *Arauz 3144* (CR); 2 Sep 2003, *Morales 9737* (INB); 16 Mar 2005, *Rodríguez 9511* (INB).

**Life form.** Epiphyte.

***Tillandsia monadelpha*** (E. Morren) Baker, J. Bot. 25: 281 (1887).

**Distribution.** Guatemala, Belize, Honduras, Nicaragua, Costa Rica, Panama, Colombia, Ecuador, Suriname, Guianas, and Venezuela.

**Representative Collections—COSTA RICA.** 16 Apr 1983, *Liesner 14312* (CR); 21 Apr 1988, *Kress 2425* (SEL); 30 May 1995, *Grant 95-2350* (CR, INB, SEL); 22 Oct 1998, *Estrada 1838* (CR); 28 Feb 2005, *Morales 12072* (INB).

**Life form.** Epiphyte.

***Tillandsia multicaulis*** Steud., Nomencl. Bot., ed. 2, 2: 688 (1841).

**Distribution.** Mexico, Guatemala, Belize, El Salvador, Honduras, Nicaragua, Costa Rica, and Panama.

**Representative Collections—COSTA RICA.** 19 Apr 1906, *Maxon 66* (NY); 21 Apr 1983, *Liesner 14528* (CR); 27 Feb 1990, *Grant 897* (SEL); 25 May 1997, *Rodríguez 2220* (CR, INB); 5 Mar 2008, *Cascante 1865* (CR).

**Life form.** Epiphyte.

**^2^*Tillandsia oerstediana*** L. B. Sm., Phytologia 13: 141 (1966).

**Distribution.** Costa Rica and Panama.

**Representative Collections—COSTA RICA.** 1 Dec 1945, *Echeverría 221* (CR); 20 Aug 1975, *Utley & Utley 2990* (CR, SCZ); 22 Jul 1990, *Luther 2818* (SEL); 8 Jan 1992, *Grant & Rundell 92-1793* (CR, INB, NY); 30 Nov 2004, *Morales 11691* (INB).

**Life form.** Epiphyte.

***Tillandsia paucifolia*** Baker, Gard. Chron., II, 10: 748 (1878).

**Distribution.** Mexico, El Salvador, Honduras, Nicaragua, Costa Rica, Venezuela, and United States of America.

**Representative Collections—COSTA RICA.** 28 Jun 1977, *Liesner 2660* (CR); 15 Jul 1992, *Grant 1975* (INB, SEL); 15 Feb 1994, *Quesada 93* (CR, INB); 15 Mar 2005, *Morales 12325* (CR, INB); 20 Feb 2007, *Vargas 2209* (INB).

**Life form.** Epiphyte.

***Tillandsia pruinosa*** Sw., Fl. Ind. Occid. 1: 594 (1797).

**Distribution.** Mexico, Guatemala, Belize, Nicaragua, Costa Rica, Panama, Colombia, Ecuador, Venezuela, Dominican Republic, Cuba, and United States of America.

**Representative Collections—COSTA RICA.** 7 Feb 1926, *Standley 47294* (INB); 1 Jul 1974, *Ocampo 680* (CR); 1 Oct 1985, *Atwood 60* (SEL); 9 Mar 1994, *Morales 2482* (CR, INB); 23 Apr 2006, *Morales 13859* (INB).

**Life form.** Epiphyte.

***Tillandsia punctulata*** Schltdl. & Cham., Linnaea 6: 53 (1831).

**Distribution.** Mexico, Guatemala, Belize, El Salvador, Honduras, Nicaragua, Costa Rica, and Panama.

**Representative Collections—COSTA RICA.** 19 Apr 1906, *Maxon 63* (NY); 19-22 Jan 1970, *Burger & Liesner 7505* (CR, NY); 29 Sep 1989, *Herrera 3599* (CR, INB); 30 Nov 1999, *Estrada 2262* (CR); 24 Feb 2008, *Santamaría 7207* (INB).

**Life form.** Epiphyte.

***Tillandsia rhomboidea*** André, Énum. Bromél.: 6 (1888).

**Syn.:** *Tillandsia acostae* Mez & Tonduz, Repert. Spec. Nov. Regni. Veg. 14: 252 (1916).

**Distribution.** Honduras, Costa Rica, Colombia, Ecuador, Peru, and Bolivia.

**Representative Collections—COSTA RICA.** 13 May 1913, *Tonduz 17891* (B, holo; CR); 3 May 1988, *Hammel 16821* (INB); Oct 1991, *Hidalgo 2* (SEL); 16 Jul 1992, *Grant 92-1983* (CR); 28 Jun 2000, *Acosta 1937* (CR, INB); 21 Aug 2005, *Morales 13321* (INB).

**Life form.** Epiphyte.

***Tillandsia schiedeana*** Steud., Nomencl. Bot., ed. 2, 2: 688 (1841).

**Distribution.** Mexico, Guatemala, Belize, El Salvador, Honduras, Nicaragua, Costa Rica, Colombia, Venezuela, Dominican Republic, Haiti, Jamaica, and Cuba.

**Representative Collections—COSTA RICA.** 1 May 1970, *Charles 1139* (NY); 12 May 1989, *Kress 2706* (SEL); 11 Oct 1995, *Jiménez 2043* (CR, INB); 15 Dec 2004, *Hammel 23489* (INB); 23 Apr 2006, *Morales 13854* (INB).

**Life form.** Epiphyte.

***Tillandsia singularis*** Mez & Wercklé, Bull. Herb. Boissier, II, 5: 103 (1905).

**Distribution.** Costa Rica, Panama, and Ecuador.

**Representative Collections—COSTA RICA.** 1908, *Wercklé 17424* (B); 11 May 1938, *Smith 572* (NY); 19 Feb 1989, *Russell 819* (CR); 18 Jan 1994, *Lépiz 127* (CR, INB); 23 Apr 2006, *Morales 13860* (INB).

**Life form.** Epiphyte.

***Tillandsia streptophylla*** Scheidw. ex C. Morren, Hort. Belge 3: 252 (1836).

**Distribution.** Mexico, Guatemala, Belize, Honduras, Nicaragua, and Costa Rica.

**Representative Collections—COSTA RICA.** 25 Mar 2005, *Morales 12453* (INB); 25 Mar 2005, *Morales 12464* (INB).

**Life form.** Epiphyte.

***Tillandsia subulifera*** Mez, Repert. Spec. Nov. Regni Veg. 16: 74 (1919).

**Distribution.** Nicaragua, Costa Rica, Panama, Colombia, and Venezuela.

**Representative Collections—COSTA RICA.** 10 Apr 1988, *Herrera 1753* (INB); 11 Dec 1990, *Herrera 4755* (INB); 7 Jan 1992, *Grant & Rundell 92-1760* (CR; INB; NY; SEL; US, n.v.); 19 Oct 1993, *Morales 1897* (INB); 19 Dec 1998, *Morales 8490* (CR, INB).

**Life form.** Epiphyte.

***Tillandsia tricolor*** Schltdl. & Cham., Linnaea 6: 54 (1831).

**Distribution.** Mexico, Guatemala, Belize, Honduras, Nicaragua, Costa Rica, and Panama.

**Representative Collections—COSTA RICA.** 15 Nov 1987, *Grayum 8453* (CR, INB); 8 Jan 1992, *Grant 92-1794* (CR; INB; NY; US, n.v.); 15 Aug 1993, *Palaci 1208* (CR, INB); 17 Jun 2002, *Boyle 6344* (CR); 5 Mar 2008, *Cascante 1858* (CR).

**Life form.** Epiphyte.

***Tillandsia usneoides*** (L.) L., Sp. Pl. ed. 2: 411 (1762).

**Distribution.** Mexico, Guatemala, Belize, El Salvador, Honduras, Nicaragua, Costa Rica, Panama, Colombia, Ecuador, Peru, Bolivia, Chile, Argentina, Uruguay, Paraguay, Brazil, Venezuela, Trinidad and Tobago, Puerto Rico, Dominican Republic, Jamaica, Cuba, and United States of America.

**Representative Collections—COSTA RICA.** 11 Jan 1893, *Tonduz 8246* (CR); 9 Sep 1978, *Burger 10982* (CR, PMA); 17 Jan 1991, *Grant 1479* (CR, INB, SEL); 18 Jan 1997, *Rodríguez 1922* (CR, INB); 27 Feb 2007, *Monro 5668* (INB).

**Life form.** Epiphyte.

***Tillandsia utriculata*** L., Sp. Pl.: 286 (1753).

**Distribution.** Mexico, Guatemala, Belize, Honduras, Nicaragua, Costa Rica, Panama, Trinidad and Tobago, Virgin Islands, Puerto Rico, Dominican Republic, Haiti, Jamaica, and United States of America.

**Representative Collections—COSTA RICA.** 16 Jul 1949, *Holm & Iltis 436* (NY); 12 Jul 1992, *Grant & Rundell 92-1937* (CR; INB; MO; NY; SEL; US, n.v.); 6 Apr 1994, *Morales 2658* (CR, INB, SEL); 14 Oct 1997, *Jiménez 2337* (CR, INB); 23 Apr 2006, *Morales 13861* (INB).

**Life form.** Epiphyte.

***Tillandsia variabilis*** Schltdl., Linnaea 18: 418 (1844).

**Distribution.** Mexico, Guatemala, Belize, Honduras, Nicaragua, Costa Rica, Panama, Colombia, Ecuador, Bolivia, Venezuela, Puerto Rico, Cuba, and United States of America.

**Representative Collections—COSTA RICA.** 13 Jul 1962, *Lee 706* (CR); 31 May 1984, *Hernández 8* (CR); 18 Feb 1991, *Till 7027* (CR); 3 Jun 1999, *Vargas 211* (INB); 23 Apr 2006, *Morales 13853* (INB).

**Life form.** Epiphyte.

***Tillandsia venusta*** Mez & Wercklé, Bull. Herb. Boissier, II, 5: 108 (1905).

**Distribution.** Costa Rica, Panama, and Ecuador.

**Representative Collections—COSTA RICA.** 30 Sep 1985, *Atwood 52* (SEL); 15 Jan 1987, *Haber 6585* (INB); 11 Mar 1991, *Richardson 82* (SEL); 4 Jun 1995, *Wilbur 64365* (CR); 16 Feb 2005, *Morales 11989* (INB).

**Life form.** Epiphyte.

***VRIESEA***

***^1^*Vriesea castaneobulbosa*** (Mez & Wercklé) J. R. Grant, J. Bromeliad Soc. 42: 14 (1992).

**Distribution.** Costa Rica.

**Representative Collections—COSTA RICA.** 23 Jul 1962, *Lee 742* (CR); Jan 1990, *Merz 659* (CR); 12 Jan 1991, *Grant 91-1363* (SEL); 8 Aug 1998, *Morales 6939* (INB); 11 Feb 2005, *Morales 11983* (INB).

**Life form.** Epiphyte.

***Vriesea chontalensis*** (Baker) L. B. Sm., Contr. U.S. Natl. Herb. 29: 518 (1951).

**Distribution.** Nicaragua, Costa Rica, Panama, Colombia, Ecuador, Peru, and Bolivia.

**Representative Collections—COSTA RICA.** *Wercklé s.n.* (B, holo; US, iso, n.v.); 1 Jan 1976, *Ocampo 1158* (CR); 6 Apr 1994, *Morales 2650* (CR, INB); 26 Apr 2001, *Mora 2011* (CR, INB); 20 Feb 2008, *Rodríguez 11767* (INB).

**Life form.** Epiphyte.

***Vriesea heliconioides*** Lindl., Ann. Bot. Syst. 3: 623 (1852).

**Distribution.** Mexico, Guatemala, Belize, Honduras, Nicaragua, Costa Rica, Panama, Colombia, Ecuador, Peru, Bolivia, Brazil, Suriname, and Venezuela.

**Representative Collections—COSTA RICA.** 6 Jul 1977, *Liesner 2993* (CR); 4 Aug 1994, *Alverson 3222* (CR, USJ); 3 Dec 1999, *Estrada 2285* (CR); 28 May 2003, *Clark 266* (SEL); 27 Jan 2007, *Morales 15366* (INB).

**Life form.** Epiphyte.

***Vriesea incurva*** (Griseb.) Read, Phytologia 16: 458 (1968).

**Distribution.** Costa Rica, Panama, Colombia, Ecuador, Peru, Bolivia, Brazil, Venezuela, and Dominican Republic.

**Representative Collections—COSTA RICA.** 1901, *Wercklé 16189* (B, holo); 6 Feb 1965, *William et al.* *28940* (SCZ); Apr 1982, *Gómez-Laurito 8146* (CR); 30 May 2006, *Rodríguez 10280* (INB); 7 Mar 2008, *Morales 16118* (INB).

**Life form.** Epiphyte.

***^1^*Vriesea lutheriana*** J. R. Grant, J. Bromeliad Soc. 42: 114 (1992).

**Distribution.** Costa Rica.

**Representative Collections—COSTA RICA.** 16 Jan 1991, *Grant 1475* (CR; SEL, iso); 12 Jun 2002, *Luther s.n.* (SEL).

**Life form.** Epiphyte.

***Vriesea monstrum*** (Mez) L. B. Sm., Phytologia 16: 81 (1968).

**Distribution.** Nicaragua, Costa Rica, Panama, Colombia, and Ecuador.

**Representative Collections—COSTA RICA.** 18 Jun 1977, *Dwyer 1495* (CR); 13 Jun 1987, *Hammel 16043* (CR); 24 Sep 1994, *Gallardo 288* (INB); 13 Oct 1995, *Cascante 824* (CR); 7 Jun 2003, *Morales 9344* (INB).

**Life form.** Epiphyte.

***WERAUHIA***

**^2^*Werauhia acuminata*** (Mez & Wercklé) J. R. Grant, Trop. Subtrop. Pflanzenwelt 91: 30 (1995).

**Distribution.** Costa Rica and Panama.

**Representative Collections—COSTA RICA.** *Wercklé 117* (B, holo); 16 Mar 1992, *Herrera 5363* (INB); 12 May 2005, *Morales 12980* (INB); 1 Mar 2007, *Solano 4104* (INB); 7 Oct 2008, *Cascante 2027* (CR).

**Life form.** Epiphyte and terrestrial.

***^1^*Werauhia ampla*** (L. B. Sm.) J. R. Grant, Trop. Subtrop. Pflanzenwelt 91: 30 (1995).

**Distribution.** Costa Rica.

**Representative Collections—COSTA RICA.** 2 Jan 1991, *Moraga 273* (INB); 6 Aug 1994, *Morales 3081* (INB, SEL); 1 Mar 1997, *Gamboa 1146* (INB); 26 Apr 2008, *Hammel 24735* (INB); 8 Jul 2008, *Cascante 1977* (CR).

**Life form.** Epiphyte and terrestrial.

****Werauhia anitana*** J. F. Morales, Novon 15: 332 (2005).

**Distribution.** Costa Rica.

**Representative Collections—COSTA RICA.** 2 Nov 2002, *Morales & Abarca 9074* (INB, holo, n.v.); 12 Dec 2002, *Morales 8919* (INB, para, n.v.); 10 Jun. 2003, *Morales & ldarraga 9432* (INB, para, n.v.).

**Life form.** Epiphyte.

***^1^*Werauhia apiculata*** (L. B. Sm.) J. R. Grant, Trop. Subtrop. Pflanzenwelt 91: 30 (1995).

**Distribution.** Costa Rica.

**Representative Collections—COSTA RICA.** 8 Apr 1992, *Marin 453* (INB); 17 Jul 1994, *Jiménez 1592* (CR, INB); 26 Aug 2000, *Luther s.n.* (SEL); 23 Apr 2006, *Morales 13856* (INB).

**Life form.** Epiphyte.

**^2^*Werauhia attenuata*** (L. B. Sm. & Pittendr.) J. R. Grant, Trop. Subtrop. Pflanzenwelt 91: 39 (1995).

**Distribution.** Costa Rica and Panama.

**Representative Collections—COSTA RICA.** 30 Apr 1980, *Meerow 1103* (SEL); 19 Apr 1988, *Kress 2408* (SEL); 6 Nov 1993, *Herrera 6642* (INB); 12 Feb 1994, *Herrera 6849* (INB).

**Life form.** Epiphyte.

***^1^*Werauhia balanophora*** (Mez) J. R. Grant, Trop. Subtrop. Pflanzenwelt 91: 39 (1995).

**Distribution.** Costa Rica.

**Representative Collections—COSTA RICA.** 1901, *Wercklé 16207* (B, holo); 12 Jul 1996, *Morales 5466* (INB, NY); 6 Mar 1997, *Navarro 687* (INB); 5 Aug 2004, *Azofeifa 111* (INB); 18 Feb 2007, *Santamaría 5710* (INB).

**Life form.** Epiphyte and terrestrial.

***^1^*Werauhia barii*** (J. F. Morales) J. F. Morales, Polibotanica 15: 110 (2003).

**Distribution.** Costa Rica.

**Representative Collections—COSTA RICA.** 5 Apr 1993, *Fernández 1052* (INB); 18 Apr 1994, *Morales 3329* (INB); 12 Jun 1996, *Morales 5387* (INB); 28 May 2005, *Morales 13105* (INB); 6 Sep 2006, *Vargas 1629* (INB).

**Life form.** Epiphyte.

**^1,2^*Werauhia bicolor*** (L. B. Sm.) J. R. Grant, Subtrop. Pflanzenwelt 91: 30 (1995).

**Distribution.** Costa Rica and Panama.

**Representative Collections—COSTA RICA.** 20 Mar 1994, *Morales 2519* (INB); 26 Jan 1996, *Morales 5103* (INB); 30 jan 2001, *Ramírez 744* (INB); 21 May 2005, *Morales 13009* (INB); 19 May 2006, *Cascante 1575* (CR).

**Life form.** Epiphyte.

**^1,2^*Werauhia bracteosa*** (Mez & Wercklé) J. R. Grant, Trop. Subtrop. Pflanzenwelt 91: 40 (1995).

**Distribution.** Costa Rica and Panama.

**Representative Collections—COSTA RICA.** 31 Mar 1995, *Morales 4898* (INB); 18 Jul 1997, *Morales 6197* (INB); 5 May 2001, *Cardelús 1383* (CR); 15 Jun 2005, *Morales 13126* (INB); 7 Sep 2005, *Acosta 3623* (INB).

**Life form.** Epiphyte.

**^1,2^*Werauhia brunei*** (Mez & Wercklé) J. R. Grant, Trop. Subtrop. Pflanzenwelt 91: 31 (1995).

**Distribution.** Costa Rica and Panama.

**Representative Collections—COSTA RICA.** 6 Apr 1993, *Herrera 6218* (INB); 18 Apr 1994, *Morales 3325* (INB); 20 Nov 1997, *Gamboa 1981* (INB); 18 Jun 2004, *Morales 10724* (INB); 22 Jun 2004, *Morales 10830* (INB).

**Life form.** Epiphyte (rarely terrestrial).

**^2^*Werauhia burgeri*** (L. B. Sm.) J. R. Grant, Phytologia 78: 121 (1995).

**Distribution.** Costa Rica and Panama.

**Representative Collections—COSTA RICA.** 23 Jan 1983, *Davidse 23227* (INB); Apr 1989, *Ferenczi 1* (SEL); 31 Mar 1996, *Alfaro 526* (INB); 3 Feb 1999, *Rodríguez 4386* (INB); 5 Mar 2008, *Morales 15982* (INB).

**Life form.** Epiphyte (rarely terrestrial).

**^1,2^*Werauhia camptoclada*** (Mez & Wercklé) J. F. Morales, Monogr. Syst. Bot. Missouri Bot. Gard. 92: 360 (2003).

**Distribution.** Costa Rica and Panama.

**Representative Collections—COSTA RICA.** Oct 1908, *Wercklé 17292* (B, holo); 14 Oct 1995, *Morales 4902* (INB); 19 Oct 2003, *Morales 10034* (INB); 28 May 2006, *Cascante 1584* (CR); 11 Nov 2008, *Cascante 2050* (CR).

**Life form.** Epiphyte.

**^2^*Werauhia capitata*** (Mez & Wercklé) J. R. Grant, Trop. Subtrop. Pflanzenwelt 91: 40 (1995).

**Distribution.** Costa Rica and Panama.

**Representative Collections—COSTA RICA.** *Wercklé 86* (B, holo); 6 Apr 1994, *Morales 2645* (INB); 20 May 1996, *Morales 5369* (INB); 7 Nov 1996, *Oconnor 30* (INB); 7 Nov 1996, *Oconnor 34* (INB).

**Life form.** Epiphyte.

**^2^*Werauhia comata*** (Mez & Wercklé) J. R. Grant, Trop. Subtrop. Pflanzenwelt 91: 40 (1995).

**Distribution.** Costa Rica and Panama.

**Representative Collections—COSTA RICA.** *Wercklé s.n.* (B, holo); 10 Feb 1992, *Ingram 1305* (INB, SEL); 19 Jul 1994, *Morales 3027* (CR, INB); 22 Jul 1994, *Quesada 128* (CR, INB); 20 Sep 2003, *Kriebel 3902* (INB).

**Life form.** Epiphyte, terrestrial, and saxicolous.

***^1^*Werauhia dodsonii*** (L. B. Sm.) J. R. Grant, Phytologia 79(3): 255 (1995).

**Distribution.** Costa Rica.

**Representative Collections—COSTA RICA.** 26 Mar 1995, *Morales 3769* (INB); 5 Jan 1996, *Morales 5048* (INB); 11 Jul 2004, *Morales 10910* (INB); 28 May 2005, *Morales 13110* (INB); 6 Mar 2008, *Morales 16047* (INB).

**Life form.** Epiphyte.

***Werauhia gladioliflora*** (H. Wendl.) J. R. Grant, Trop. Subtrop. Pflanzenwelt 91: 31 (1995).

**Distribution.** Mexico, Belize, Nicaragua, Costa Rica, Panama, Colombia, and Ecuador.

**Representative Collections—COSTA RICA.** 27 May 1968, *Burger 5391* (CR, NY); 25 Feb 1991, *Till 7110* (CR); 8 Jan 1992, *Grant 1774* (CR, INB, SEL); 27 Jun 2001, *Chaves 1214* (INB); 6 Mar 2008, *Morales 16057* (INB).

**Life form.** Epiphyte and terrestrial (rarely saxicolous).

***Werauhia graminifolia*** (Mez & Wercklé) J. R. Grant, Trop. Subtrop. Pflanzenwelt 91: 32 (1995).

**Distribution.** Nicaragua, Costa Rica, Panama, and Ecuador.

**Representative Collections—COSTA RICA.** 29 Dec 1974, *Taylor 17733* (SCZ); 14 Jan 1984, *Gómez 20792* (CR); 18 Feb 1990, *Grant 793* (INB); 1 Apr 1997, *Rodríguez 2079* (INB); 10 Jan 2000, *Alfaro 2659* (INB).

**Life form.** Epiphyte (rarely terrestrial).

***Werauhia greenbergii*** (Utley) J. R. Grant, Trop. Subtrop. Pflanzenwelt 91: 42 (1995).

**Distribution.** Costa Rica, Panama, and Ecuador.

**Representative Collections—COSTA RICA.** 27 Jan 1976, *Utley 3741* (CR); 10 Nov 1993, *Bello 5405* (CR, INB); 17 Apr 1994, *Morales 2699* (CR, INB); 18 Feb 1995, *Morales 4884* (INB); 20 Mar 2005, *Santamaría 1152* (INB).

**Life form.** Epiphyte, terrestrial, and saxicolous.

***^1^*Werauhia haberi*** (J. F. Morales) J. F. Morales, Polibotanica 15: 110 (2003).

**Distribution.** Costa Rica.

**Representative Collections—COSTA RICA.** 23 Dec 1988, *Haber 8947* (INB).

**Life form.** Epiphyte.

***^1^*Werauhia hainesiorum*** (L. B. Sm.) J. R. Grant, Trop. Subtrop. Pflanzenwelt 91: 42 (1995).

**Distribution.** Costa Rica.

**Representative Collections—COSTA RICA.** 20 Feb 1994, *Morales 3337* (INB); 18 Apr 1994, *Morales 3330* (INB); 22 May 1994, *Morales 3363* (INB).

**Life form.** Epiphyte.

***Werauhia hygrometrica*** (André) J. R. Grant, Trop. Subtrop. Pflanzenwelt 91: 42 (1995).

**Distribution.** Guatemala, Honduras, Nicaragua, Costa Rica, Panama, Colombia, and Ecuador.

**Representative Collections—COSTA RICA.** Apr 1975, *Velick 1* (SEL); 27 Sep 1988, *Ingram 252* (SEL, CR, NY); 22 Jul 1994, *Lépiz 510* (CR, INB); 6 Apr 2005, *Soto 695* (INB, NY); 22 Feb 2007, *Rodríguez 10692* (INB).

**Life form.** Epiphyte, terrestrial, and saxicolous.

**^2^*Werauhia insignis*** (Mez) W. Till, Barfuss & M. R. Samuel, J. Bromeliad Soc. 54: 13 (2004).

**Syn.:** *Tillandsia insignis* (Mez) L. B. Sm. & Pittendr., J. Wash. Acad. Sci. 43: 402 (1953).

**Distribution.** Costa Rica and Panama.

**Representative Collections—COSTA RICA.** 1 Jan 1922, *Brenes 3595* (CR); 20 Mar 1959, *Charles 89* (NY); 20 Dec 1982, *Zuchowski 608* (SEL); 10 Mar 2001, *Mora 1901* (CR, INB, NY); 16 Jun 2005, *Morales 13169* (INB).

**Life form.** Epiphyte, terrestrial, and saxicolous.

**^1,2^*Werauhia kathyae*** (Utley) J. R. Grant, Trop. Subtrop. Pflanzenwelt 91: 42 (1995).

**Distribution.** Costa Rica and Panama.

**Representative Collections—COSTA RICA.** 24 Apr 1983, *Liesner 14676* (INB); 22 Feb 1990, *Grant 824* (INB, SEL); 21 Jul 1994, *Morales 3055* (CR, INB); 20 Feb 1999, *Vargas 157* (INB); 17 Jun 2005, *Morales 13220* (INB).

**Life form.** Epiphyte (rarely terrestrial).

***Werauhia kupperiana*** (Suess.) J. R. Grant, Trop. Subtrop. Pflanzenwelt 91: 32 (1995).

**Distribution.** Nicaragua, Costa Rica, Panama, Colombia, and Ecuador.

**Representative Collections—COSTA RICA.** 15 Sep 1986, *Davidse 31283* (INB); 19 Oct 1989, *Funk 10599* (INB); 3 Dec 1993, *Morales 2127* (CR, INB, SEL); 17 Apr 1994, *Morales 2703* (CR, INB, SEL); 18 Nov 1996, *Oconnor 202* (INB).

**Life form.** Epiphyte and terrestrial.

**^2^*Werauhia latissima*** (Mez & Wercklé) J. R. Grant, Trop. Subtrop. Pflanzenwelt 91: 43 (1995).

**Distribution.** Costa Rica and Panama.

**Representative Collections—COSTA RICA.** *Wercklé s.n.* (B, holo, n.v); *Wercklé 82* (B, n.v.); *Wercklé 8* (US, n.v.); 10 Apr 2005, *Soto 773* (INB).

**Life form.** Epiphyte (rarely terrestrial).

**^2^*Werauhia laxa*** (Mez & Wercklé) J. R. Grant, Trop. Subtrop. Pflanzenwelt 91: 43 (1995).

**Distribution.** Costa Rica and Panama.

**Representative Collections—COSTA RICA.** *Wercklé 90* (B, holo); 26 Feb 1975, *Utley 1857* (CR, INB, NY); 12 Feb 1992, *Ingram 1325* (INB, SEL); 20 Jul 1996, *Morales 5533* (INB); 16 Apr 2004, *Solano 978* (INB).

**Life form.** Epiphyte, terrestrial, and saxicolous.

**^2^*Werauhia leucophylla*** (L. B. Sm.) J. R. Grant, Trop. Subtrop. Pflanzenwelt 91: 43 (1995).

**Distribution.** Costa Rica and Panama.

**Representative Collections—COSTA RICA.** 29 Jun 1972, *Utley 401* (CR); 13 Sep 1978, *Burger & Antonio 11024* (CR, SCZ); 22 Jul 1990, *Luther 2804* (SEL); 20 Mar 1994, *Morales 2520* (INB); 25 Sep 2005, *Solano 2705* (INB).

**Life form.** Epiphyte (occasionally terrestrial).

***^1^*Werauhia luis-gomezii*** (Utley) J. R. Grant, Trop. Subtrop. Pflanzenwelt 91: 43 (1995).

**Distribution.** Costa Rica.

**Representative Collections—COSTA RICA.** 11 Jul 1996, *Morales 5460* (INB); 1 Apr 1997, *Quesada 1965* (CR, INB); 21 May 2005, *Morales 13008* (INB); 15 Jun 2005, *Morales 13133* (INB).

**Life form.** Epiphyte (occasionally terrestrial).

***^1^*Werauhia lyman-smithii*** (Utley) J. R. Grant, Trop. Subtrop. Pflanzenwelt 91: 43 (1995).

**Distribution.** Costa Rica.

**Representative Collections—COSTA RICA.** 3 Oct 1993, *Morales 1825* (INB); 29 Mar 1994, *Morales 2614* (INB); 21 Feb 1995, *Morales 3492* (INB); 19 Mar 1996, *Morales 5330* (INB); 7 Dec 2004, *Morales 11742* (INB).

**Life form.** Epiphyte.

**^1,2^*Werauhia macrantha*** (Mez & Wercklé) J. R. Grant, Phytologia 78: 121 (1995).

**Distribution.** Costa Rica and Panama.

**Representative Collections—COSTA RICA.** *Wercklé s.n.* (B, holo); 31 Mar 1995, *Morales 3824* (INB); 28 Jan 1996, *Morales 5201* (INB).

**Life form.** Epiphyte.

**^1,2^*Werauhia macrochlamys*** (Mez & Wercklé) J. F. Morales, Lundiana 4(1): 65 (2003).

**Distribution.** Costa Rica and Panama.

**Representative Collections—COSTA RICA.** *Wercklé 115* (B, holo); 21 Jul 1994, *Morales 3047* (INB); 17 Oct 1996, *Moraga 775* (CR, INB); 22 Nov 2004, *Soto 346* (INB); 27 Jul 2005, *Acosta 3524* (INB).

**Life form.** Epiphyte (occasionally terrestrial).

**^2^*Werauhia marnier-lapostollei*** (L. B. Sm.) J. R. Grant, Trop. Subtrop. Pflanzenwelt 91: 33 (1995).

**Distribution.** Costa Rica and Panama.

**Representative Collections—COSTA RICA.** 21 Apr 1994, *Lépiz 305* (INB); 20 Apr 1995, *Morales 3881* (INB); 3 Apr 1997, *Morales 6159* (INB); 4 Apr 2002, *Arauz 3250* (CR); 7 Apr 2005, *Morales 12782* (INB).

**Life form.** Epiphyte.

****Werauhia moralesii*** H. Luther, Brittonia 54(4): 281 (2002 publ. 2003).

**Syn.:** *Werauhia clandestina* J.F. Morales, Polibotanica 15: 110 (2003).

**Distribution.** Costa Rica.

**Representative Collections—COSTA RICA.** 22 Jul 1990, *Luther 2801* (MO, SEL); 17 Apr 1994, *Morales 2704* (INB).

**Life form.** Epiphyte, terrestrial, and saxicolous.

**Note.** According to The Plant List (www.theplantlist.org) “this name is unresolved”. The species was originally described as *Vriesea simulans* J.F. Morales in Novon 9: 404 (1999), which is a homonym of *Vriesea simulans* Leme in J. Bromeliad Soc. 47(4): 169 (1997).

***Werauhia nephrolepis*** (L. B. Sm. & Pittendr.) J. R. Grant, Trop. Subtrop. Pflanzenwelt 91: 44 (1995).

**Distribution.** Guatemala, El Salvador, Honduras, Nicaragua, Costa Rica, and Panama.

**Representative Collections—COSTA RICA.** 1901, *Wercklé 16201* (B); 27 Feb 1987, *Grayum 8097* (INB); 19 Sep 1997, *Rodríguez 2479* (INB); 6 Apr 2005, *Soto 694* (INB, NY); 5 Feb 2008, *Cascante 1842* (CR).

**Life form.** Epiphyte (occasionally terrestrial).

**^1,2^*Werauhia notata*** (L. B. Sm. & Pittendr.) J. R. Grant, Trop. Subtrop. Pflanzenwelt 91: 44 (1995).

**Distribution.** Costa Rica and Panama.

**Representative Collections—COSTA RICA.** 4 Oct 1898, *Tonduz 12526* (B, holo; CR); 14 Jan 1992, *Grant 1870* (CR, INB); 26 Mar 1995, *Morales 3771* (INB); 8 Mar 2000, *Acosta 557* (CR, INB); 7 Oct 2008, *Cascante 2022* (CR).

**Life form.** Epiphyte and terrestrial.

***Werauhia ororiensis*** (Mez) J. R. Grant, Trop. Subtrop. Pflanzenwelt 91: 44 (1995).

**Distribution.** Costa Rica, Panama, and Ecuador.

**Representative Collections—COSTA RICA.** 2 Jun 1890, *Tonduz 2159* (B, CR); 14 Apr 1968, *Rodríguez & Sevilla 1143* (USJ); 5 Mar 1976, *Utley & Utley 4238* (SCZ); 12 Jan 1992, *Grant 1842* (INB, SEL); 16 Jun 2005, *Morales 13186* (INB).

**Life form.** Epiphyte and terrestrial.

***^1^*Werauhia osaensis*** (J. F. Morales) J. F. Morales, Polibotanica 15: 110 (2003).

**Distribution.** Costa Rica.

**Representative Collections—COSTA RICA.** 26 Aug 1990, *Herrera 4144* (INB); 4 Sep 1993, *Aguilar 2227* (INB); 15 Mar 1995, *Mora 83* (INB); 13 May 2001, *Morales 8069* (INB); 28 Feb 2005, *Morales 12071* (INB).

**Life form.** Epiphyte.

**^1,2^*Werauhia paniculata*** (Mez & Wercklé) J. R. Grant, Trop. Subtrop. Pflanzenwelt 91: 45 (1995).

**Distribution.** Costa Rica and Panama.

**Representative Collections—COSTA RICA.** *Wercklé 55* (B, lecto); 25 Jun 1976, *Utley 5196* (CR); 17 Jun 1994, *Morales 2937* (INB); 30 Jul 2001, *Acosta 3127* (INB); 17 Jun 2005, *Morales 13216* (INB).

**Life form.** Epiphyte (occasionally terrestrial).

***Werauhia pedicellata*** (Mez & Wercklé) J. R. Grant, Trop. Subtrop. Pflanzenwelt 91: 47 (1995).

**Distribution.** Honduras, Nicaragua, Costa Rica, and Panama.

**Representative Collections—COSTA RICA.** *Wercklé 116* (B, holo; GH, iso, n.v.); 28 Apr 1980, *Meerow 1037* (SEL); 6 Apr 1994, *Morales 2641* (CR, INB, SEL); 15 May 2004, *Kriebel 4641* (INB); 10 Mar 2008, *Cascante 1883* (CR).

**Life form.** Epiphyte (occasionally terrestrial).

**^2^*Werauhia picta*** (Mez & Wercklé) J. R. Grant, Trop. Subtrop. Pflanzenwelt 91: 47 (1995).

**Distribution.** Costa Rica and Panama.

**Representative Collections—COSTA RICA.** *Wercklé 119* (B, lecto); May- Jun 1976, *Utley & Utley 4979* (CR).

**Life form.** Epiphyte.

**^2^*Werauhia pittieri*** (Mez) J. R. Grant, Trop. Subtrop. Pflanzenwelt 91: 33 (1995).

**Distribution.** Costa Rica and Panama.

**Representative Collections—COSTA RICA.** Apr 1898, *Tonduz 12229* (B, holo, n.v.; US, iso, n.v.); 6 Apr 1995, *Aguilar 3884* (INB); 24 Jul 1997, *Rodríguez 2410* (INB); 5 Jul 2000, *Morales 7288* (CR, INB); 9 Dec 2004, *Morales 11873* (INB).

**Life form.** Epiphyte.

***Werauhia ringens*** (Griseb.) J. R. Grant, Trop. Subtrop. Pflanzenwelt 91: 35 (1995).

**Distribution.** Honduras, Nicaragua, Costa Rica, Panama, Colombia, Ecuador, Dominican Republic, and Haiti.

**Representative Collections—COSTA RICA.** 12 Mar 1993, *Luther s.n.* (SEL); 23 Nov 1994, *Morales 3224* (CR, INB); 17 May 1995, *Morales 4210* (INB); 15 Mar 2004, *Morales 10320* (INB).

**Life form.** Epiphyte.

**^2^*Werauhia rubra*** (Mez & Wercklé) J. R. Grant, Trop. Subtrop. Pflanzenwelt 91: 47 (1995).

**Distribution.** Costa Rica and Panama.

**Representative Collections—COSTA RICA.** *Wercklé 100* (B, lecto); 11 May 1975, *Utley 2486* (CR); 8 Sep 1984, *Davidse 28704* (CR); 21 Feb 1995, *Morales 3493* (CR, INB, NY); 29 Aug 2000, *Rodríguez 6346* (INB).

**Life form.** Epiphyte and terrestrial.

***^1^*Werauhia rugosa*** (Mez & Wercklé) J. R. Grant, Phytologia 79(3): 255 (1995).

**Distribution.** Costa Rica.

**Representative Collections—COSTA RICA.** *Wercklé s.n.* (B); 29 Apr 1980, *Meerow et al. 1073* (SEL); 6 Aug 1994, *Morales 3077* (INB).

**Life form.** Epiphyte (rarely terrestrial).

***Werauhia sanguinolenta*** (Linden ex Cogn. & Marchal) J. R. Grant, Trop. Subtrop. Pflanzenwelt 91: 35 (1995).

**Distribution.** Honduras, Nicaragua, Costa Rica, Panama, Colombia, Ecuador, Peru, Bolivia, Brazil, Venezuela, Dominican Republic, and Jamaica.

**Representative Collections—COSTA RICA.** 5 Jan 1992, *Grant 1729* (CR, INB, SEL); 3 Dec 1993, *Morales 2136* (CR, INB, SEL); 27 Jul 1995, *Lépiz 612* (CR, INB); 13 Nov 1998, *Estrada 1933* (CR); 11 Apr 2005, *Morales 12752* (INB).

**Life form.** Epiphyte (occasionally terrestrial and saxicolous).

**^1,2^*Werauhia singuliflora*** (Mez & Wercklé) J. R. Grant, Trop. Subtrop. Pflanzenwelt 91: 48 (1995).

**Distribution.** Costa Rica and Panama.

**Representative Collections—COSTA RICA.** *Wercklé 89* (B, holo); 31 Jul 1982, *Gómez-Laurito 8914* (INB); 31 Mar 1995, *Morales 4899* (INB); 19 Mar 1996, *Morales 5336* (INB); 12 May 2005, *Morales 12959* (INB).

**Life form.** Epiphyte.

**^2^*Werauhia stenophylla*** (Mez & Wercklé) J. R. Grant

Trop. Subtrop. Pflanzenwelt 91: 48 (1995).

**Distribution.** Costa Rica and Panama.

**Representative Collections—COSTA RICA.** *Wercklé 112* (B, holo); 12-17 Dec 1969, *Burger & Liesner 6734* (SCZ); 22 Feb 1990, *Grant 844* (INB); 12 Feb 1992, *Ingram 1323* (SEL).

**Life form.** Epiphyte (occasionally terrestrial).

***Werauhia subsecunda*** (Wittm.) J. R. Grant, Trop. Subtrop. Pflanzenwelt 91: 35 (1995).

**Distribution.** Costa Rica, Panama, and Colombia.

**Representative Collections—COSTA RICA.** *Wercklé 118* (B, holo); 27 Apr 1980, *Meerow 1010* (SEL); 14 Jan 1992, *Grant 1869* (CR, INB); 9 Dec 2004, *Morales 11911* (INB); 23 Apr 2008, *Cascante 1923* (CR).

**Life form.** Epiphyte.

***^1^*Werauhia tiquirensis*** (J. F. Morales) J. F. Morales, Polibotanica 15: 110 (2003).

**Distribution.** Costa Rica.

**Representative Collections—COSTA RICA.** 22 Apr 1995, *Morales 3984* (INB); 23 Jun 1995, *Morales 4462* (INB); 13 May 2001, *Morales 8024* (INB); 6 Apr 2005, *Morales 12506* (INB).

**Life form**. Epiphyte and terrestrial.

***^1^*Werauhia tonduziana*** (L. B. Sm.) J. R. Grant, Trop. Subtrop. Pflanzenwelt 91: 35 (1995).

**Distribution.** Costa Rica.

**Representative Collections—COSTA RICA.** 15 Jan 1991, *Grant 1438* (INB); 9 Nov 1992, *Ingram 1701* (CR, SEL); 10 May 2005, *Morales 12873* (INB); 19 May 2006, *Cascante 1574* (CR); 26 May 2008, *Hammel 24778* (INB).

**Life form.** Epiphyte (rarely terrestrial).

***Werauhia umbrosa*** (L. B. Sm.) J. R. Grant, Trop. Subtrop. Pflanzenwelt 91: 48 (1995).

**Distribution.** Costa Rica, Panama, and Colombia.

**Representative Collections—COSTA RICA.** 20 Apr 1993, *Ingram 1845* (INB, SEL); 5 Jan 1997, *González 1495* (INB); 13 Apr 2005, *Santamaría 1654* (INB); 11 May 2005, *Morales 12904* (INB); 9 Aug 2007, *Soto 1697* (INB).

**Life form.** Epiphyte and terrestrial.

***^1^*Werauhia uxoris*** (Utley) J. R. Grant, Trop. Subtrop. Pflanzenwelt 91: 48 (1995).

**Distribution.** Costa Rica.

**Representative Collections—COSTA RICA.** 1 May 1994, *Morales 3333* (INB); 11 Jul 1996, *Morales 5457* (INB); 5 Sep 1996, *Morales 5790* (INB); 21 May 2005, *Morales 13031* (INB); 16 Jun 2005, *Morales 13185* (INB).

**Life form.** Epiphyte.

***^1^*Werauhia vietoris*** (Utley) J. R. Grant, Trop. Subtrop. Pflanzenwelt 91: 48 (1995).

**Distribution.** Costa Rica.

**Representative Collections—COSTA RICA.** 5 Mar 1980, *Meerow 1117* (SEL); 28 Mar 1994, *Morales 2598* (INB); 10 Apr 1994, *Morales 2674* (INB); 19 Jul 2000, *Rodríguez 5981* (INB).

**Life form.** Epiphyte.

***Werauhia viridiflora*** (Regel) J. R. Grant, Trop. Subtrop. Pflanzenwelt 91: 38 (1995).

**Distribution.** Mexico, Guatemala, Belize, Honduras, Nicaragua, Costa Rica, Panama, Colombia, Ecuador, and Venezuela.

**Representative Collections—COSTA RICA.** *Wercklé 66* (B, lecto); 15 Feb 1990, *Grant 90-766* (SEL); 16 Jul 1992, *Ingram 1539* (CR, SEL); 28 Mar 2003, *Morales 9265* (INB); 23 Feb 2008, *Rodríguez 11907* (INB).

**Life form.** Epiphyte.

**^1,2^*Werauhia viridis*** (Mez & Wercklé) J. R. Grant, Trop. Subtrop. Pflanzenwelt 91: 49 (1995).

**Distribution.** Costa Rica and Panama.

**Representative Collections—COSTA RICA.** *Wercklé s.n.* (B, holo; US, iso, n.v.); 1 Apr 1976, *Utley 4421* (INB); 29 Mar 1994, *Morales 2613* (INB); 3 Feb 1995, *Morales 3434* (INB); 19 Mar 1996, *Morales 5326* (INB).

**Life form.** Epiphyte.

***Werauhia vittata*** (Mez & Wercklé) J. R. Grant, Trop. Subtrop. Pflanzenwelt 91: 38 (1995).

**Distribution.** Belize, Nicaragua, Costa Rica, Panama, and Ecuador.

**Representative Collections—COSTA RICA.** *Wercklé 79* (B, holo); 12 Jan 1981, *Waggoner s.n.* (SEL); 22 Feb 1990, *Grant 848* (INB); 10 May 1995, *Morales 4077* (INB); 1 May 2004, *Morales 10567* (INB).

**Life form.** Epiphyte.

***^1^*Werauhia vulcanicola*** (J. F. Morales) J. F. Morales, Polibotanica 15: 110 (2003).

**Distribution.** Costa Rica.

**Representative Collections—COSTA RICA.** 9 Jan 1993, *Morales 2237* (INB); 15 Aug 1996, *Morales 5704* (INB); 12 Apr 2005, *Morales 12780* (INB).

**Life form.** Terrestrial.

***Werauhia werckleana*** (Mez) J. R. Grant, Trop. Subtrop. Pflanzenwelt 91: 38 (1995).

**Distribution.** Mexico, Guatemala, Belize, El Salvador, Honduras, Nicaragua, Costa Rica, and Panama.

**Representative Collections—COSTA RICA.** 1 Jan 1901, *Wercklé 16210* (B, holo; US, iso, n.v.); 14 Jan 1992, *Grant 92-1865* (CR, INB, SEL); 22 Jan 1994, *Morales 2287* (INB, NY, SEL); 8 Jan 1996, *Penneys 982* (CR, INB); 26 Apr 2008, *Hammel 24736* (INB).

**Life form.** Epiphyte (occasionally terrestrial).

***Werauhia williamsii*** (L. B. Sm.) J. R. Grant, Trop. Subtrop. Pflanzenwelt 91: 49 (1995).

**Distribution.** Nicaragua, Costa Rica, and Panama.

**Representative Collections—COSTA RICA.** 9 Aug 1971, *Burger & Burger 7950* (SCZ); 5 May 1980, *Meerow 1177* (SEL); 11 Jul 1996, *Morales 5459* (INB); 7 Jun 2003, *Alfaro 4446* (INB); 7 Sep 2006, *Vargas 1634* (INB).

**Life form.** Epiphyte (occasionally terrestrial).
